# Evaluation of a Stepped-Care eHealth HIV Prevention Program for Diverse Adolescent Men Who Have Sex With Men: Protocol for a Hybrid Type 1 Effectiveness Implementation Trial of SMART

**DOI:** 10.2196/19701

**Published:** 2020-08-11

**Authors:** Brian Mustanski, David A Moskowitz, Kevin O Moran, Michael E Newcomb, Kathryn Macapagal, Carlos Rodriguez-Díaz, H Jonathon Rendina, Eric B Laber, Dennis H Li, Margaret Matson, Ali J Talan, Cynthia Cabral

**Affiliations:** 1 Institute for Sexual and Gender Minority Health and Wellbeing Northwestern University Chicago, IL United States; 2 Department of Medical Social Sciences Feinberg School of Medicine Northwestern University Chicago, IL United States; 3 Department of Prevention and Community Health Milken Institute School of Public Health George Washington University Washington, DC United States; 4 Graduate School of Public Health Medical Sciences Campus University of Puerto Rico San Juan Puerto Rico; 5 Department of Psychology Hunter College of the City University of New York (CUNY) New York, NY United States; 6 Department of Statistics North Carolina State University Raleigh, NC United States; 7 Department of Psychiatry and Behavioral Sciences Feinberg School of Medicine Northwestern University Chicago, IL United States

**Keywords:** HIV prevention, eHealth, adolescents, men who have sex with men, implementation science, mobile phone

## Abstract

**Background:**

Adolescent men who have sex with men (AMSM), aged 13 to 18 years, account for more than 80% of teen HIV occurrences. Despite this disproportionate burden, there is a conspicuous lack of evidence-based HIV prevention programs. Implementation issues are critical as traditional HIV prevention delivery channels (eg, community-based organizations, schools) have significant access limitations for AMSM. As such, eHealth interventions, such as our proposed SMART program, represent an excellent modality for delivering AMSM-specific intervention material where youth *are*.

**Objective:**

This randomized trial aimed to test the effectiveness of the SMART program in reducing condom-less anal sex and increasing condom self-efficacy, condom use intentions, and HIV testing for AMSM. We also plan to test whether SMART has differential effectiveness across important subgroups of AMSM based on race and ethnicity, urban versus rural residence, age, socioeconomic status, and participation in an English versus a Spanish version of SMART.

**Methods:**

Using a sequential multiple assignment randomized trial design, we will evaluate the impact of a stepped-care package of increasingly intensive eHealth interventions (ie, the universal, information-based SMART Sex Ed; the more intensive, selective SMART Squad; and a higher cost, indicated SMART Sessions). All intervention content is available in English and Spanish. Participants are recruited primarily from social media sources using paid and unpaid advertisements.

**Results:**

The trial has enrolled 1285 AMSM aged 13 to 18 years, with a target enrollment of 1878. Recruitment concluded in June 2020. Participants were recruited from 49 US states as well as Puerto Rico and the District of Columbia. Assessments of intervention outcomes at 3, 6, 9, and 12 months are ongoing.

**Conclusions:**

SMART is the first web-based program for AMSM to take a stepped-care approach to sexual education and HIV prevention. This design indicates that SMART delivers resources to all adolescents, but more costly treatments (eg, video chat counseling in SMART Sessions) are conserved for individuals who need them the most. SMART has the potential to reach AMSM to provide them with a sex-positive curriculum that empowers them with the information, motivation, and skills to make better health choices.

**Trial Registration:**

ClinicalTrials.gov Identifier NCT03511131; https://clinicaltrials.gov/ct2/show/NCT03511131

**International Registered Report Identifier (IRRID):**

DERR1-10.2196/19701

## Introduction

### Background

Adolescent men who have sex with men (AMSM) in the United States account for 83% of all new HIV occurrences among those aged 13 to 19 years. The majority of these cases (86%) are among racial and ethnic minority youth [1]. Despite these health disparities, there have been no prevention interventions targeted specifically at this population [2]. Current evidence-based HIV prevention programs focus primarily on adults and heterosexual youth [3]. However, as the issues affecting sexual health decisions among AMSM are unique (eg, access to affirming care) [4,5], interventions should be designed with their needs in mind to ensure that the content resonates with them. Moreover, prevention programs need to be responsive to racial and ethnic minority AMSM who experience reduced access to HIV or sexually transmitted infection (STI) prevention services [6,7] and, as a corollary, increased HIV incidence [1]. eHealth interventions represent a critical modality for delivering AMSM-specific intervention material where youth *are*, considering that 97% of adolescents across all races and income levels are on the web every day [8]. Combining web-based recruitment with intervention delivery across a range of devices could overcome many access barriers to the engagement of AMSM in HIV prevention. Here, we describe a hybrid type 1 effectiveness-implementation protocol [9] aimed at testing the SMART Program’s effectiveness and informing future implementation as a service. Our study uses a sequential multiple assignment randomized trial [10,11] to examine the effectiveness of each component of SMART, which consists of three eHealth HIV prevention interventions.

### HIV Acquisition Risk in AMSM

AMSM are the most at risk for HIV infection compared with all other subgroups of adolescents because of specific risk factors [1]. Some of these factors that contribute to inconsistent condom use are common to AMSM and adult men who have sex with men (MSM) alike, such as substance use before or during sex [12], familiarity with partners [13], and negative affective states such as loneliness or depression [14,15]. Other factors are more unique to AMSM, such as access to and cost of condoms [16], inconsistent sexual health education [17,18], sexual inexperience [14], sex with older partners [19,20], and underage use of sexual networking apps for adults [21]. Adult MSM have had increasing access to pre-exposure prophylaxis (PrEP) since it was approved by the Food and Drug Administration in 2012 [22]; however, adolescents, for whom PrEP was only recently approved in mid-2018, report extremely low uptake [23-25]. Knowledge about PrEP, self-efficacy to access it, fear of lack of parental support or punishment, state-level requirements of parental consent to use it, and regimen upkeep (including quarterly HIV testing) have been cited as reasons for this difference between AMSM and adult MSM [23-26].

Despite engagement in HIV transmission risk behaviors and representing a large proportion of adolescent HIV diagnoses, AMSM have not achieved sufficiently high rates of HIV testing. A recent study found that only 23% of AMSM aged 13 to 18 years reported testing at least once for HIV in their lifetime [27]. Adolescents, especially AMSM, fail to test because of fears about family or pediatrician and health care provider judgments, being closeted, or being afraid of testing positive [28-31]. Lack of adolescent-friendly testing sites, barriers to transportation, and fear of being seen by friends also contribute to reduced testing rates [32,33]. Even among those who test, failure to repeatedly test or establish a testing regimen has been noted [27]. This suggests that existing sexual health education may be insufficient, lack relevance or depth, or simply may not be reaching AMSM [17,18].

### AMSM HIV Prevention Programs and eHealth

The delivery of relevant HIV prevention measures for AMSM through traditional channels (eg, schools, parents) is extremely restricted. For example, the vast majority of school-based sexual education programs do not address the needs of sexual and gender minority adolescents [17,34], and many schools have explicit policies prohibiting the discussion of homosexuality [35,36]. Parent-child HIV prevention programs are efficacious in reducing sexual risk among heterosexual youth [37-41]. However, even in an era of greater acceptance, many parents of the most at-risk AMSM [42,43] reject their teen’s sexual identity [44-46] or refuse to discuss same sex behaviors [47]. eHealth, or the use of electronic technologies to promote health [48], has the potential to circumvent these barriers. It represents a relatively anonymous manner to easily access knowledge that may otherwise be stigmatizing or endangering if sought using in-person methods (eg, information on sexual orientation, engaging in anal sex, PrEP) [49-51]. Considering that 95% of teens report having a smartphone and 45% report being on the web constantly [8], web-based programs that provide sexual health information regardless of location have excellent potential to reach AMSM.

Several noteworthy eHealth interventions have already targeted AMSM. Queer Sex Ed (QSE) was tested in 2013 with AMSM aged 16 to 20 years and young adult MSM [52]. This web-based program emphasized sexual health as more than just the absence of disease and included information on healthy romantic relationships, having pleasurable sexual experiences, and acceptance of one’s sexual orientation and gender identity. Intervention content also explained HIV or STI transmission and how to acquire and use condoms. A total of 15 of the 17 primary outcome measures significantly improved from baseline to posttest (2 weeks later), including the knowledge of safer sex practices. Guy2Guy (G2G) was a text message–based intervention that provided 14- to 18-year-old sexually experienced and inexperienced sexual minority males with text messages on safer sex, having sex in the context of a relationship, and HIV testing [53]. G2G also paired participants with each other so they could practice program skills and provide social support. A 2014 efficacy trial showed that participants in the intervention arm of G2G reported a three-fold increase in HIV testing relative to those in the information-only control group; however, condom-less anal sex did not differ between the groups.

### Hybrid Type 1 Effectiveness Implementation Design

These previous eHealth interventions [52,53] suggest that web-based programs can be effective for sexual minority adolescents. However, none of these programs have systematically examined factors critical to their real-world implementation. The traditional, stepwise pipeline of intervention development to implementation is estimated to take 17 years and takes only a fraction of interventions from research to practice [54,55]. Hybrid designs serve to accelerate this process by concurrently examining effectiveness and implementation outcomes, thereby shortening the time needed to study both [9]. Our adaptation of pre-existing effective interventions also improves this acceleration by reducing the likelihood of null findings and increasing the ease of scale-out. As we are using a hybrid type 1 effectiveness implementation design, the primary aim of our study is to establish evidence of effectiveness for SMART; however, we will also gather preliminary implementation data to inform future selection of implementation strategies to scale our program. Specifically, we are utilizing the Reach, Effectiveness, Adoption, Implementation, and Maintenance (RE-AIM) outcomes framework [56-58] to measure implementation outcomes by focusing on reach, effectiveness, cost, ongoing delivery, and program sustainment. In addition, we are drawing from the Consolidated Framework for Implementation Research (CFIR) [59] to assess contextual determinants that may impact future implementation (eg, implementation readiness, barriers, facilitators, and ease of integration). These data can provide convincing information for decision makers about how to implement SMART once effectiveness testing ends [9,60].

### Objectives

SMART is a suite of stepped-care interventions, following the Institute of Medicine’s prevention model [61,62]. This model suggests increasing the intensity of prevention techniques according to risk factors or specific risks exhibited by a given population. The first step in SMART is a low-cost universal intervention offered to all participants regardless of HIV risk (ie, SMART Sex Ed, SSE). The second more intensive selective intervention (ie, SMART Squad) is offered to those who report HIV risk intentions or behaviors following SSE. Finally, a higher cost indicated intervention (ie, SMART Sessions) is designed for those who continue to report HIV risk intentions or behavior following the two previous interventions. We are testing for individual intervention and cumulative intervention effectiveness at reducing condom-less anal sex and increasing condom use intentions, self-efficacy, and HIV testing among AMSM participants. Additionally, we are testing whether SMART has differential effectiveness across subgroups of AMSM based on race and ethnicity, urban versus rural residence, age, socioeconomic status, and preference for an English versus a Spanish version of the intervention. Our use of a hybrid type 1 design simultaneously allows us to collect data that will provide critical insight into factors that may impact SMART’s real-world implementation. In the following section, we describe the protocol for all 3 components of SMART.

## Methods

### Study Design

**Figure 1:**
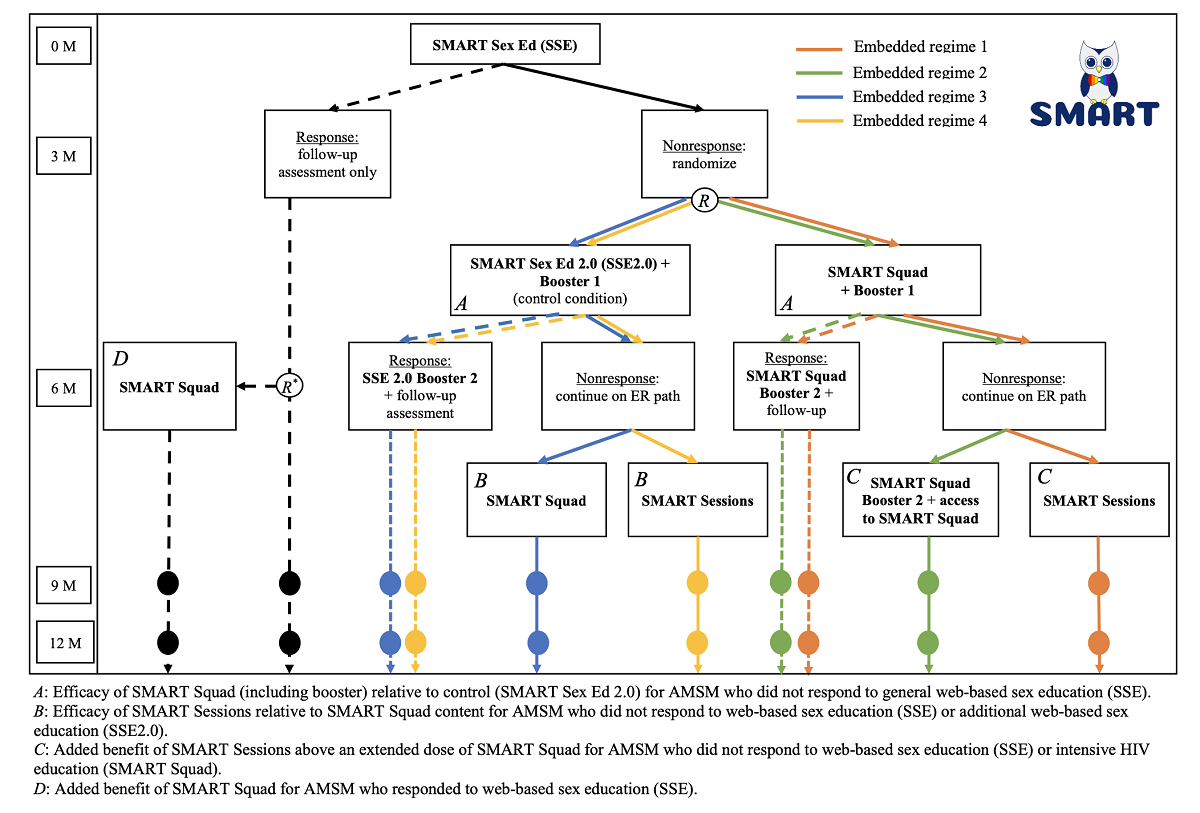
SMART participant intervention progression. Survey assessments are conducted at baseline (0 M), 3 months (3 M), 6 months (6 M), 9 months (9 M), and 12 months (12 M). An embedded regime is the path or sequence of specified interventions to which a participant may be randomized. The circled letter “R” refers to the point at which participants are randomized to an embedded regime, or in the case of responders to SSE (see R* ), either follow-up only or access to SMART Squad after the 6-month assessment. Dashed embedded regime paths represent responder pathways. AMSM: adolescent men who sleep with men; SSE: SMART Sex Ed.

This study uses a hybrid type 1, sequential multiple assignment randomized trial [10,11,63] evaluating the impact of a package of increasingly intensive, stepped-care interventions (Figure 1). The advantage of using a sequential design is that it can help determine which of a wide variety of intervention strategies (or combinations therein) will be best suited to a given individual, thus maximizing efficacy. Previous versions of SMART intervention steps have already shown evidence of efficacy with diverse young adult MSM [52,64,65] and were further developmentally and linguistically adapted to accommodate the unique social experiences and health barriers of English- and Spanish-speaking adolescents in this study [66]. All participants received the universally relevant SSE intervention at baseline. Response to the intervention, as defined in the section below, will be measured at the 3-month follow-up assessment. Those who respond to SSE will be randomized to receive either SMART Squad or a follow-up only condition. Those who do not respond to SSE will be randomized to receive 1 of 4 treatment packages, 2 of which include the control condition, SMART Sex Ed2.0 (SSE2.0). As shown in Figure 1, these treatment packages represent pathways a participant could take through the trial contingent on their responder status and are thus termed *embedded regimes* [63,67,68].

Embedded regime 1 assigns the participant to the selective SMART Squad initially; if the participant is a nonresponder at 6 months, then it assigns the participant to SMART Sessions. If the participant is a responder at 6 months, then it assigns the participant to SMART Squad Booster 2 and follow-up.

Embedded regime 2 assigns the participant to a selective SMART Squad initially; if the participant is a nonresponder at 6 months, then it assigns the participant to SMART Squad Booster 2 and continued access to SMART Squad. If the participant is a responder at 6 months, then it assigns the participant to SMART Squad Booster 2 and follow-up.

Embedded regime 3 assigns the participant to receive SSE2.0 initially; if the participant is a nonresponder at 6 months, then it assigns the participant to SMART Squad. If the participant is a responder at 6 months, then it assigns the participant to SSE2.0 Booster 2 and follow-up.

Embedded regime 4 assigns the participant to receive SSE2.0 initially; if the participant is a nonresponder at 6 months, then it assigns the participant to SMART Sessions. If the participant is a responder at 6 months, then it assigns the participant to SSE2.0 Booster 2 and follow-up.

Randomization by embedded regimes is mathematically equivalent to running separate randomizations at each stage; however, from an implementation perspective, randomization to embedded regimes is often easier with clinical trial software (ie, REDCap, Research Electronic Data Capture), especially when randomization is stratified or subject to other constraints.

### Defining Response to the Intervention

At each time point, the response threshold is defined as meeting each of these 3 criteria: (1) 100% condom use, if the participant is sexually active in the assessment period, (2) intentions for condom use during all instances of penetrative sex (regardless of reported sexual activity), and (3) reporting a high degree of self-efficacy for achieving condom use during all instances of penetrative sex (regardless of sexual reported activity). Condom use intentions are assessed using the 11-item Condom Use Intentions Scale [69,70]. This scale measures the likelihood of condom use under varying situations. An example item is, “If you have a boyfriend, how likely would you be to use condoms with him?” Each item has a 4-option response scale ranging from *very unlikely* to *very likely*. The self-efficacy of condom use is assessed using the Condom Use Self-Efficacy Scale [69,70]. This scale measures perceived confidence in being able to engage in safer sex practices under varying circumstances. An example item is, “How confident are you that you would be able to refuse to have anal sex without a condom?” Each item has a 7-option scale ranging from *not at all confident* to *extremely confident*. A mean score of 4 for condom use intentions and a mean score of 7 for condom self-efficacy would indicate 100% condom use intentions and self-efficacy.

Calibrating condom use intention and self-efficacy thresholds specifically for SMART was necessary to establish values that were meaningful in terms of prevention impact. The threshold calibrations also needed to be stringent enough to err on the side of escalation to a more intense intervention. Overall, the calibration process sought to establish the optimal treatment sequence in this stepped-care design. To do this, we tested the condom use intentions and self-efficacy items on a sample of 204 AMSM who were enrolled in a separate study to establish the distribution of values for these scales. As the aim was to select *a priori* thresholds for SMART’s sample that would reflect a 90% nonresponse rate before SSE, we calculated the 90th percentile values from the 204 AMSM for condom use intentions and self-efficacy. A 90% nonresponse rate was required and calculated from an initial power analysis to ensure that the second- and third-tier interventions would be sufficiently powered. These values then became the initial responsiveness threshold for condom use intentions (ie, scores >3.76) and self-efficacy (ie, scores >6.50) for SMART. When enrollment into SMART opened in April 2018, study statisticians monitored the first 58 participants enrolled to verify the reliability of the condom use intention and self-efficacy thresholds between actual SMART participants and the previous sample of 204 AMSM. Most (51/58, 88%) of the first 58 SMART participants did not exceed the score set for responding. This finding was deemed acceptably close to 90% to retain those criteria.

As such, SMART participants are considered responsive to any intervention if they report all of the following: (1) 100% condom use, if sexually active in the assessment period, (2) a condom use intentions score >3.76 (ie, *very likely* to use condom), and (3) a self-efficacy score >6.50 (ie, *extremely confiden*t at using condoms). It is important to understand that with this type of research design, *responsive* is the threshold for participants to be considered for randomization to the next intervention step; this is not the same value that defines the success of the intervention in terms of the effectiveness outcomes.

### Inclusion and Exclusion Criteria

Potential participants are eligible for this study according to the following inclusion criteria: (1) they were assigned male at birth, (2) they identify as a sexual minority (ie, report their sexual orientation as gay, bisexual, queer, lesbian, or pansexual) or report attraction to cisgender males, (3) they report an HIV negative or unknown HIV status, (4) they have engaged in sexual contact with another person (defined as having touched another person’s genitals or performed oral, vaginal, or anal sex), (5) are between the ages of 13 and 18 years (inclusive), (6) have access to or use the internet, (7) are able to read and speak English or Spanish at a sixth grade level or better, (8) and reside in the United States, including Puerto Rico, Guam, and the US Virgin Islands. Current gender identity was not an inclusion or exclusion criterion. Those assigned male at birth could identify as any gender identity (eg, transgender, nonbinary, genderqueer, genderfluid) provided they met the 8 inclusion criteria. Those identifying as intersex or assigned female at birth were excluded to comply with the trail’s specific aim to curtail HIV spread in AMSM.

Potential participants are ineligible if the study staff identify discrepancies between the eligibility screener and the baseline assessment. Such discrepancies may include reporting 2 different ages on the screener and baseline assessment, reporting different zip codes or locations, and/or not reporting lifetime sexual contact on the baseline assessment.

### Recruitment, Eligibility Screening, and Enrollment Into SMART

**Figure 2:**
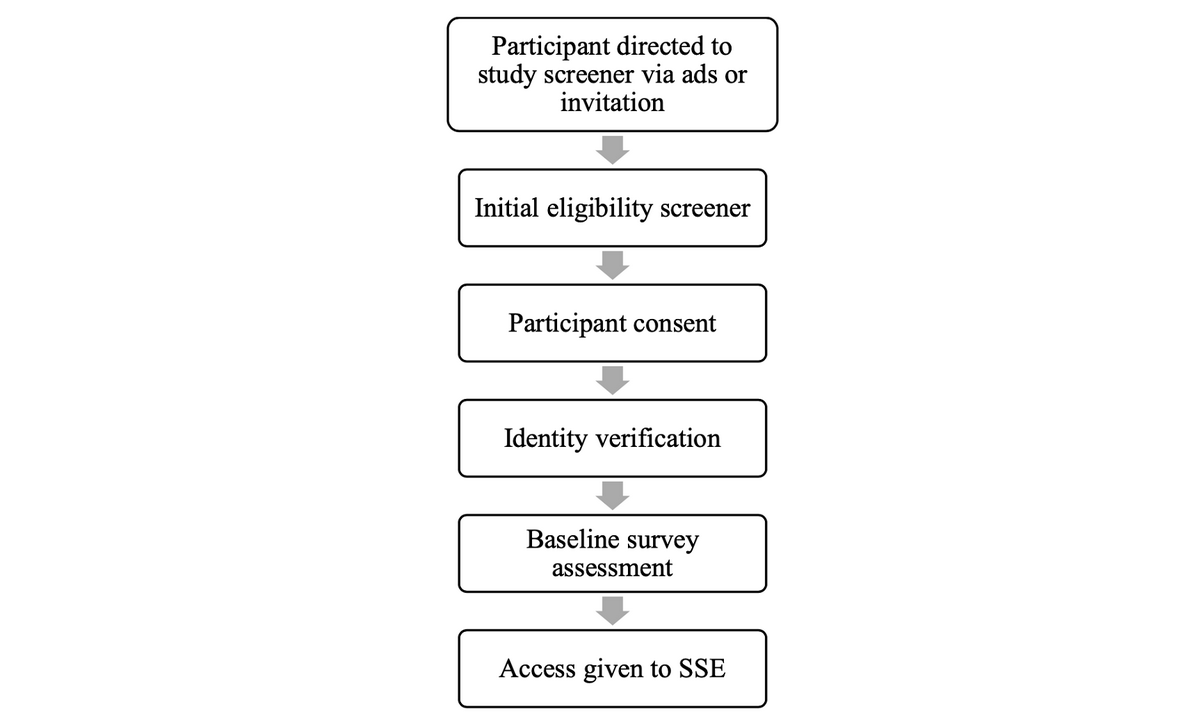
Participant entrance into SMART: from advertisement to enrollment. SSE: SMART Sex Ed.

English- and Spanish-speaking AMSM are recruited using paid advertising on social media (eg, Instagram, Facebook) and through active web-based engagement using geospatial dating apps and other social media outlets (eg, Reddit, Tumblr). Advertisements, posts, and direct messages direct potential participants to a brief web-based eligibility survey, available in English and Spanish. Participants who complete the survey in English are given access to the English-only version of SMART. Those who complete it in Spanish are given access to the Spanish-only version of SMART, in which all study consent, communications or reminders, intervention content, and assessments are provided in Spanish. Figure 2 displays participant flow from advertisement to enrollment. All study surveys are administered via REDCap [71]. The Northwestern University Institutional Review Board granted SMART a waiver of signed documentation of informed consent or assent as well as a waiver of parental permission for participants under 18 years. Participants are routed to a consent page with 4 decisional capacity questions, which assess their comprehension of study tasks, risks, and benefits, as well as how to exit the study [72]. They also submit a username for study staff approval on the consent page. Usernames cannot have any personally identifying information (eg, name, email). If they provide consent, study staff email and/or text prospective participants to set up a video chat to verify participant identity, review the study tasks, and answer any of their questions. During this 5-min video chat, AMSM are also asked to explain back to study staff what they will be asked to do as a SMART participant. Finally, if a participant has submitted a username with personally identifying information, the study staff will work with the participant to revise the username while on the video chat.

Once the video chat is complete, participants are sent their web-based baseline assessment survey, which has all pertinent primary and secondary study measures. Completion of the baseline assessment triggers an automatic email inviting the participant to login to SMART by going to the website, resetting their password using their username, and then logging in to access the first tier of the intervention. All participants, regardless of demographic characteristics or responses to their baseline assessment, are given access to SSE.

### Randomization of Treatment Arms

Nonresponders to SSE are randomized to 1 of 4 embedded regimes, which determines the interventions that a participant receives and the order in which they occur. This assignment is performed using stratified block randomization [73]. Through stratification, we avoid an imbalance of prespecified factors that may be related to the primary outcomes and/or to the intervention delivery itself. We randomized within 8 strata comprising all combinations of the following 3 binary factors: language preference (English or Spanish), rurality (living in an urban or rural zip code), and lifetime anal sex experience (any or none). Within each stratum, embedded regimes were assigned using a permuted block design, with blocks of size 4. This ensures that at any point during the study, each embedded regime assignment is protected against large imbalances in language preference, rurality, and sexual experience. The R package *blockrand* [74] was used to create the randomization allocation table.

We selected these stratifying factors for several reasons. The SMART program is delivered in either English or Spanish depending on participant preference. Although the content is identical across language delivery, cultural factors may lead AMSM to be differentially responsive to the content and style of the interventions [75-78]. As such, we wish to ensure that English and Spanish speakers are equally represented in each randomization assignment. Rurality is included as a stratifying factor because of potential differences in lived experiences when comparing rural AMSM with nonrural AMSM. Rural AMSM may feel less comfortable coming out, have less family support, and have less access to the lesbian, gay, bisexual, transgender, queer (LGBTQ) community organizations and providers who are knowledgeable about LGBTQ health and HIV [16,79,80]. Rural residence is assessed by categorizing participant-reported zip codes into rural-urban commuting area codes [81]; zip codes with 30% or more of their workers going to a census-defined *Urbanized Area* were considered urban and all others were considered rural. Finally, a lifetime penetrative sexual experience is included to account for differential HIV risk among those who have engaged in anal sex with a male and those who have not. Additionally, elements of the intervention content may be differentially applicable to those who have had penetrative sex based on their lived experiences.

### Treatment Conditions

All tiers of SMART were built from the information-motivation-behavioral (IMB) skills model for HIV prevention [82]. This model suggests that individuals are likely to enact behaviors if they are knowledgeable or informed about the behavior, motivated to enact it, and have the corresponding skills to enact the behavior. In adult MSM [83-85], this model has shown that individuals with accurate HIV knowledge, sufficient motivation (eg, fear, HIV vulnerability), and know where to screen for HIV are more likely to complete HIV testing. Similarly, IMB constructs have been associated with condom use consistency and PrEP use among MSM [64,69,86-89]. These studies indicate that knowledge is necessary but not always sufficient to move MSM toward prevention and testing behaviors, and individuals who report higher levels of the 3 IMB components tend to be more likely to engage in HIV prevention and testing. SMART builds on this evidence by taking a tiered approach to HIV prevention messaging to AMSM, that is, for some, merely providing basic information on HIV prevention will be sufficient to improve condom use. SSE (tier 1) is therefore built as an information-only intervention to which all participants will be granted access. For those who do not respond to HIV-related information, providing situational and contextual HIV prevention motivations, and training in HIV behavioral skills to prevent transmission can increase behavioral enactment. As such, we built SMART Squad (tier 2) and SMART Sessions (tier 3) to provide all 3 theoretical constructs from IMB to participants who continue to report inconsistent condom use intentions and behaviors.

#### SMART Sex Ed

**Figure 3:**
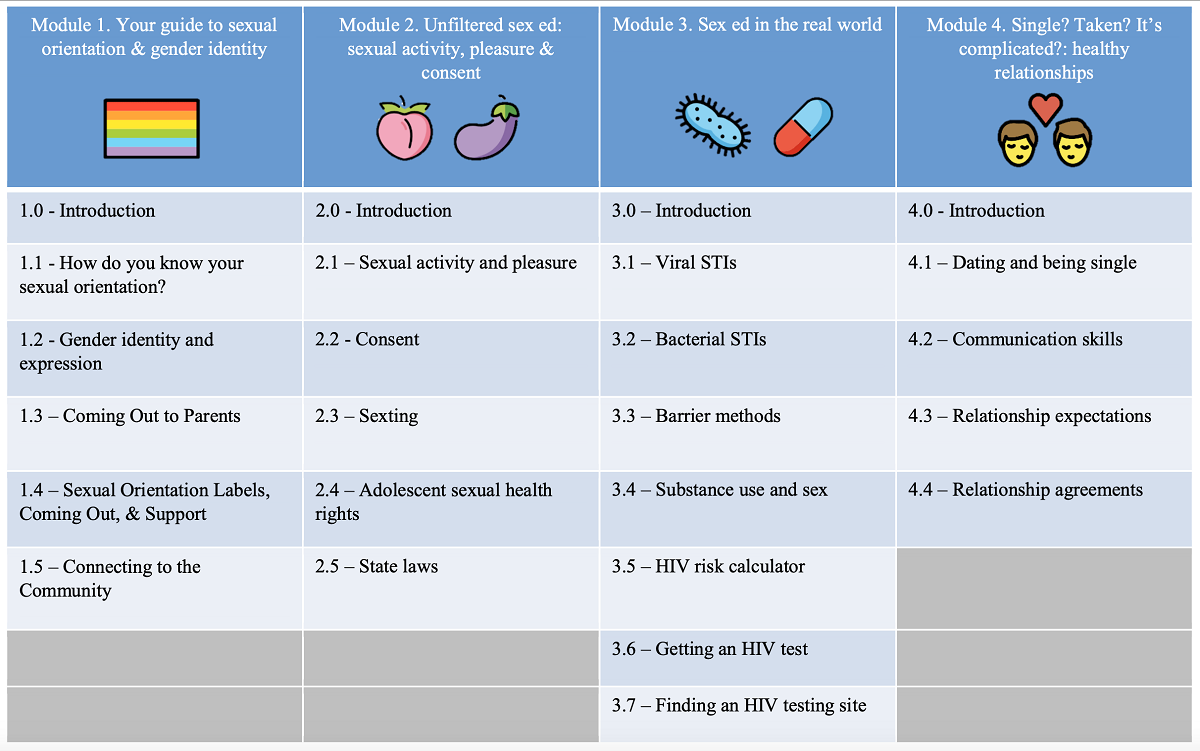
Overview of the 4 SMART Sex Ed modules. STI: sexually transmitted infection.

SSE represents the first-tier intervention for SMART. It is exclusively informational in nature and was adapted from an intervention previously tested on LGBTQ youth showing preliminary efficacy (ie, QSE) [52]. As part of the adaptation process [90], core sexual health competencies and learning objectives from the Centers for Disease Control and Prevention (CDC) [91] and Sexuality Information and Education Council of the United States [92] were incorporated and, if necessary, were updated to suit a sexual minority audience (eg, coming out strategies). We assembled a diverse, standing web-based youth advisory council of AMSM (13- to 18-year olds) to review our adapted content and answer questions about the relevance of information we were considering incorporating. Members of the council acted as an asynchronous focus group and were compensated monthly for their time [93]. Besides ensuring that SSE content would resonate with AMSM, this focus group allowed community member stakeholders (ie, AMSM) to participate in the intervention creation. SSE contains 4 modules that participants can navigate in their preferred order (Figure 3). Media assets used across the modules include full-page scroll screens (resembling social media feeds), slideshows with narration recorded using near-peer voice actors, videos, games, quizzes, and graphic interchange format images. Emojis are liberally used to make topics and lessons more tangible to participants who commonly use emojis in peer-to-peer web-based communication to discuss sexual behavior. *SMART Facts* are used to segue between modules. They describe LGBTQ historical moments (eg, the Stonewall riots) and LGBTQ racial and ethnic identity intersectionality (eg, pictures and a historic description of the Native Hawaiian LGBTQ experience). All modules end with a content quiz for participants, which helps them identify areas they may want to review. When participants select an incorrect response, they are given messaging that explains why their choice is incorrect and why another answer may be the better option.

Figure 3 gives a visual overview of modules and their subsections. The first module covers sexual orientation and gender identity in detail. The differences between the 2 are identified, with both being further framed as continuous social constructs (eg, what transgender means relative to nonbinary, what distinguishes someone identifying as gay from pansexual, and why people describe their sexual or gender identity on a continuum). *Coming out* is explained and participants are given tips for how to disclose sexual orientation or gender identity to family. Finally, community resources and LGBTQ-friendly organizations are suggested for participants who may want more specific help regarding understanding their sexual or gender identities.

The second module explores sexual behaviors (eg, receptive anal sex), including how to minimize discomfort and maximize pleasure. Detailed discussions of sexual consent are provided as well as an explanation of the sexual health rights of adolescents (eg, a state-by-state map explaining laws about sexual health testing and access to services without parental consent).

The third and longest module introduces participants to biological and behavioral sexual health. Although traditional topics such as differences between bacterial and viral STIs are discussed in detail, this section elaborates on the sexual health needs of AMSM. For example, the role of lubrication during anal sex is explained as a protective factor when used with condoms, PrEP is described, relative differences in sexual risk behaviors are visually depicted using an HIV risk calculator, and how to find a friendly LGBTQ-oriented HIV or STI testing site is provided.

Finally, participants were introduced to the topic of healthy relationships in the fourth module. Different relationship configurations are described (eg, being single, dating, being in multiple relationships) and the differences between monogamy and nonmonogamy are explained. Suggestions for enacting direct communication about relationship expectations are given.

#### SMART Squad

**Figure 4:**
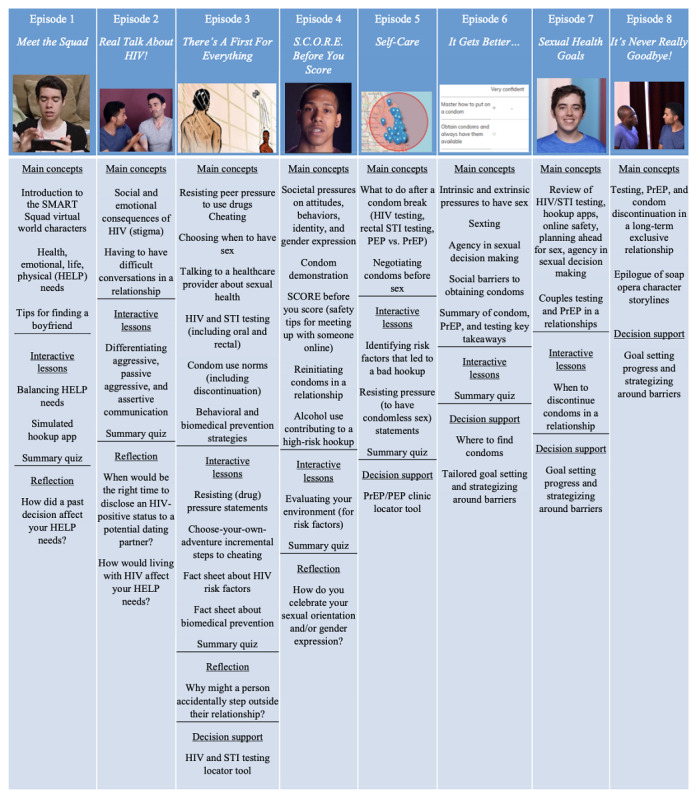
Overview of the 8 SMART Squad episodes: main concepts and active learning components. Main concepts refer to learning objectives or topics covered by episodes. Interactive lessons refer to activities that provide positive motivations and skills for sexual health. Reflection refers to open-ended questions asked of participants as an activity within an episode. Decision support refers to skills-based activities to identify solutions to health barriers. PEP: postexposure prophylaxis; PrEP: pre-exposure prophylaxis; STI: sexually transmitted infection.

SMART Squad represents the experimental second-tier intervention for SMART. Differing in many ways from the SSE, SMART Squad focuses on improving participants’ motivations to concentrate on their sexual health and behavioral skills to enact protective measures to prevent HIV or STIs. This intervention was adapted from *Keep it Up!*, a CDC best-evidence effective intervention previously tested on young adult MSM [64,94] using intervention mapping as a systematic approach [66]. All the adapted content, including all scripted videos, were reviewed by our web-based youth advisory council. SMART Squad contains 6 episodes and 2 booster episodes; the first booster is delivered 1 month after the completion of episode 6, and the second is delivered 3 months after the completion of episode 6. Participants were forced to break for 8 hours between episodes 3 and 4. Figure 4 [66] describes the main concepts and active learning components within each episode.

The educational modalities used are different in SMART Squad relative to SSE. This intervention relies on a scripted video soap opera delivered across the episodes. It features interactive activities that encourage participants to reflect on their motivations and help them build behavioral skills. SMART Squad also has a forum where participants can post asynchronous messages to each other under topics like *breaking the mold and being yourself*, *best/worst dates you’ve had*, and *parents/guardians*. The forum has a topic called, *ask the Sexpert*, where participants can post questions, which are directly answered by study staff who provide health education but not medical advice. Finally, after episode 6, participants complete a goal-setting activity where they select 3 prevention or risk-reduction goals to accomplish in the next 1 and 3 months. These goals include, but are not limited to getting an HIV test, obtaining a condom, using condoms during every sexual encounter, and talking to a health care provider about PrEP. Once selected, the activity helps users think through how to overcome likely barriers using suggested strategies to achieve the goals. Briefly, SMART Squad encourages participants to consider their own sexual identity, sexual health, and psychological challenges, and identify the best ways to overcome them.

The video soap opera follows 4 main characters who are in geographically different high schools across the United States. These characters meet each other in a web-based space called *SMART Squad* and become fast friends. They share with each other different sexual orientation, sexual behavior, and relationship problems they encounter in their daily lives and ask each other (and other characters) for advice. Participants follow their storylines as the characters make healthy and unhealthy decisions and learn from their successes and failures. In addition to the video soap opera, there are activities that conceptually and visually align with the videos. For example, one of the video characters is about to have sex for the first time. His older partner is pressuring him to have condom-less sex, and the character does not know how to respond. At that moment in the video, an activity pops up for participants to help the character by rating potential *condom comebacks* as weak or strong. The strongest response from the activity is spoken by the character when the video restarts, enabling successful condom use.

These examples demonstrate how changes in motivations and behavioral skills are enacted throughout SMART Squad using interconnected videos and activities. Peer norms and tension for change are instilled through the storylines, and skill-building exercises support self–re-evaluation, stimulus control, and reinforcement management. In terms of specific content, episode 1 focuses on health, emotional, life, and physical needs, as well as tips for dating. Episode 2 delves into the social and emotional consequences of HIV infection (eg, stigma, disclosure) and shows how to have difficult conversations in a relationship (eg, discussing infidelity with a main partner). Episode 3 is the longest episode and covers how to resist peer pressure to use drugs, how and when to choose to have anal sex with a partner, how to talk to health care providers about sexual health, HIV or STI testing, condom use norms, and behavioral and biomedical prevention strategies (eg, PrEP). Episode 4 introduces participants to societal pressures around gender norms, features a condom demonstration, itemizes the steps to consider before meeting an unfamiliar or anonymous partner for sex, describes how to reinitiate condoms into a relationship, and shows how alcohol and drug use contributes to sexual risk behaviors. Episode 5 outlines the steps to take if condoms are not used or if the condom breaks (ie, postexposure prophylaxis). It also shows how to negotiate condom use before sex with a partner. Finally, episode 6 concludes the main intervention by covering the intrinsic and extrinsic pressures to have sex, sexting, control/agency surrounding sex with partners, and overcoming barriers to obtaining condoms. This last episode also has the characters reiterate the overall importance of condoms, PrEP, and consistent HIV or STI testing.

The 2 boosters (ie, episodes 7 and 8) do not introduce new concepts but reinforce main themes from the first 6 episodes, continue the storyline of the characters several months later, and conclude the plotlines. The 2 boosters also serve as check-ins for participants regarding the goals they made after episode 6. Participants provide feedback whether they accomplished their goals. If they have, they are asked to select a new goal. If they have not, they are asked to provide reasons for not completing the goal, and then SMART Squad provides additional strategies to help.

#### SMART Sex Ed 2.0 (Control Condition)

SSE2.0 represents the second-tier control arm for SMART. One of the main hypotheses driving the design of this study was that some AMSM would need more than information to reduce their HIV risk. SMART Squad reflects this by addressing motivations and behavioral skills. The logical control condition for SMART Squad would be the continuation of an information-based intervention but without HIV prevention motivational and skill-building content. SSE2.0 was developed as an expanded version of the SSE (with 6 modules and 2 boosters). One key difference between SSE and SSE2.0 is that participants must go through the SSE2.0 modules in a specific order to match how participants advance through SMART Squad. Participants start with a module that reviews what sex and sexual behaviors are, the importance of pleasure, and health communication. Module 2 provides additional facts about STIs that were not covered in SSE. Module 3 is exclusively about HIV and shows the epidemiology of the disease, including which groups are more at risk of infection. Module 4 outlines the different types of barrier methods to prevent HIV or STI infection (eg, traditional condoms, internal or receptive condoms, lube, dental dams). Module 5 discusses PrEP, postexposure prophylaxis, and treatment as prevention. The final module in SSE2.0 provides an overview of HIV testing and HIV treatment. The first booster, which opens 1 month after the completion of module 6, discusses drugs or alcohol and their relationship with sexual consent and sexual risk taking. The second booster, which opens 3 months after the completion of module 6, identifies HIV risk factors and the steps participants can take to avoid those factors.

#### SMART Sessions

SMART Sessions represent the third-tier intervention for SMART. Similar to SMART Squad, this intervention focuses on motivations, skill building, and goal setting for participants. However, where SMART Squad uses an automated web platform, SMART Sessions rely on one-on-one video chat motivational interviewing (MI) counseling between participants and SMART coaches. This program was adapted from the Young Men’s Health Project, an effective intervention previously tested on young adult MSM [65]. SMART Sessions are delivered by clinical professionals with postgraduate training in counseling or psychology. All coaches receive extensive training in MI techniques and conduct mock sessions to be cleared to deliver the intervention to SMART participants [95]. Coaches receive weekly individual and group supervision by a licensed clinical psychologist to ensure quality delivery of MI principles. Participants who are randomized to SMART Sessions participate in 3 to 4 video chat sessions over the course of 4 to 6 weeks via Skype or FaceTime. The number of sessions is determined by the coach, based on whether the participant reports engagement in condom-less sex and/or is a strong candidate for PrEP. Video chats last between 20 and 45 min, on average, and participants remain with the same coach for all their sessions. The minimum time for being considered a completed session is 15 min.

The 4 sessions focus on increasing motivation to engage in safer sex behaviors, including using condoms during sexual intercourse, receiving an HIV or STI test or creating a routine around testing, and PrEP use. The first session begins with introductions, an explanation of the overall timeline and content of SMART Sessions, limits to confidentiality, and a *priorities* activity. This activity asks the participant to list the most important priorities in their lives and asks about the following 5 priorities and how they might fit into the priorities that the participant has already listed: family, independence, sexuality, school, and health. The coach then asks the participant to select their top 3 priorities from the list and discuss how these priorities might be related to the decisions that they make around sexual health. The purpose of this activity is to consider how HIV prevention may fit in with the participant’s broader goals and values and to serve as a jump-off point for discussing the participant’s sexual health practices. At this point, the coach collaborates with the participant regarding what topic they would like to explore first—HIV prevention or HIV testing. Using MI strategies, the coach works with the participant to identify changes that they may want to make to their sexual health plan and encourages the participant to brainstorm ways in which they may begin to make those changes. Participants are asked to take into account past successes that they might have had regarding sexual health. The first session ends with a summary of their discussions and scheduling the second session. The second session mimics the first, but focuses on whichever topic was not previously addressed (HIV prevention or HIV testing). By the end of the first 2 sessions, the participant and coach would have discussed both topic areas, identified moments for potential behavior change regarding prevention and testing, and developed potential sexual health goals for consideration.

The third session takes a different direction by focusing on PrEP education and PrEP navigation. The session begins with a review of sessions 1 and 2 and a recount of any successes or failures surrounding HIV prevention and/or testing. Following this, the coach provides the participant with a brief educational overview of PrEP, including its usefulness and navigation options (ie, who prescribes it, where to find providers). Together, the coach and participant explore ideas about whether PrEP might be a right fit or identify future milestones for the participant that may signify it might be right to start PrEP (ie, becoming sexually active, having multiple sex partners). If PrEP is a good choice for the participant, the coach and participant discuss strategies and goals to move the participant toward PrEP acquisition and use. The session ends with a review of PrEP and the coach answering any additional questions from the participant. If this is the final session, there is also a review of all the material covered in the previous sessions, a discussion regarding what sexual health resources are available to the participant and the coach saying goodbye to the participant. The fourth session, for those designated in advance (ie, those actively engaging in condom-less anal sex), begins with the participant describing progress made since initiating SMART Sessions. The coach spends time highlighting the changes in the participant’s thinking and describes the progress that the coach perceives the participant has made. Together, they discuss obstacles to past change and steps to take toward future change regarding HIV prevention, testing, and, if applicable, PrEP uptake. The coach works with the participant to identify commitment statements, which the participant should consider before enacting risk behaviors, if applicable. Goals are finalized, and any concluding questions or concerns are answered before this last session is completed.

If a participant reports a safety concern (eg, they are experiencing suicidal or homicidal ideation, they are currently being abused or maltreated by a caregiver), the coach will conduct a safety assessment with the participant to determine the level of risk involved. The coach will then consult with their supervisor to determine whether further action, including mandatory reporting, needs to be taken.

All sessions conducted are audio-recorded by the coaches. Weekly supervision occurs within SMART Sessions, in which coaches’ sessions are reviewed and analyzed by a clinical psychologist with advanced proficiency in MI and Motivational Interviewing Treatment Integrity (MITI) 4.2.1 coding [96]. Coaches are provided with guidance on how to enhance their delivery of MI techniques. SMART Session recordings (20%) are coded for MI fidelity using the MITI coding system [96]. Sessions are individually coded by a group of trained MITI coders.

### Study Assessments and Other Measures

Whether participants graduate from 1 intervention tier to the next is contingent on how they answer the previously described condom use attitudinal questions (ie, condom intention and self-efficacy items) and the behaviors they report. To prevent participant anticipatory effects (ie, misreporting with the intent to receive more or less treatment), they are not told the criteria for intervention response. Participants complete self-reported questionnaires at all follow-up time points (ie, 3, 6, 9, and 12 months post-SSE). They are compensated US $25 for completing each assessment, for a total of up to US $125 per participant. Figure 1 shows the flow of events for participants, and Table 1 provides a list of the primary and secondary outcomes by assessment time point.

**Table 1:** Primary, secondary, and other outcome measures: operationalization and schedule.

Constructs	Measures and operationalization	Measurement schedules				
		Baseline	3 months	6 months	9 months	12 months
Sexual risk (P^a^)	Condom-less anal sex partners as well as sex acts with the most recent 3 partners [97]	x^b^	x	x	x	x
Condom use intentions and self-efficacy (P)	Condom Use Intentions Scale–11 items; Condom Use Self-Efficacy Scale–5 items [69,70]	x	x	x	x	x
HIV testing (P)	Self-reported history of testing for HIV in the previous 3 months [98]	x	x	x	x	x
HIV knowledge (S^c^)	Knowledge of HIV transmission and prevention [99]	x	x	—^d^	—	x
Motivation and behavioral skills (S)	Motivation (eg, motivation to become safer), social norms (eg, partners’, friends’, or family members’ opinions about condom use), and behavioral skills (eg, negotiating condom use) [69]	x	x	x	x	x
Condom errors (S)	Adapted condom errors questionnaire–15 items [100]	x	x	x	x	x
Substance use (O^e^)	Alcohol use disorders identification test, cannabis use disorders identification test, past 3-month use of illicit drugs [101]	x	x	x	x	x
PrEP^f^ (O)	PrEP knowledge, current and past 3-month PrEP use, PrEP adherence, motivation to start PrEP, and reasons for discontinuation [102,103]	x	x	x	x	x

^a^P: primary outcomes.

^b^Measure is assessed.

^c^S: secondary outcomes.

^d^Measure is not assessed because the first-tier intervention was the only one to focus on HIV information. As such, HIV knowledge was assessed before and after this intervention (baseline and 3-month assessments), as well as the final time point (12-months) to assess knowledge retention.

^e^O: other outcomes.

^f^PrEP: pre-exposure prophylaxis.

### Implementation Science

As this is a hybrid type 1 implementation trial, we are measuring additional constructs from the CFIR [59] and RE-AIM models (eg, reach, adoption, integration) [56,58,104] to help improve future implementation and dissemination of SMART. Internal accounting for costs, recruitment activities, staff and investigator effort, resources, and stakeholder attitudes have occurred during the intervention development and the ongoing trial. With respect to enrolled participants, we actively and passively collect key data on their interactions with the different interventions, attitudes toward them, and the amount of time they spend within them. Within interventions, participants can give a *thumbs-up* or *thumbs-down* on each activity and overall module or episode; we also provide an open-ended textbox to allow them to provide feedback about an activity, a section, or an overall module or episode. We follow all interventions with an adapted version of an HIV intervention acceptability and tolerability battery [105], which includes open- and closed-ended items. This battery assesses participant engagement, impact, usefulness, and usability per intervention. Additionally, for SMART Sessions, we assess participants’ perceived quality of interaction with SMART coaches [106]. Finally, after participants have graduated from the randomized controlled trial and completed their 12-month survey, participants are invited to complete a 30-min exit interview with the study staff. These participants explain their overall attitudes toward SMART (as a suite of interventions), identify areas for overall improvement, and provide suggestions for ways to publicly implement the program.

The SMART platform has sophisticated backend software to collect analytics or *paradata*. Time spent on every page of intervention content is measured per participant per intervention. This allows us to assess the overall time for each of the interventions, whether participants are rushing through or taking too long to complete any interventions and whether participants are engaged with specific pieces of any given intervention relative to others. SMART Sessions have different passive measurements that are collected by the SMART coaches. These include the duration and frequency of a session, session notes, and overall impressions of the session; the MITI coding previously mentioned also serves as implementation data.

### Analytic Plan

Our primary aim is to compare the differential effects of 2 web-based interventions, the active treatment of SMART Squad and the control condition of SSE2.0 among nonresponders to SSE in terms of the 3 primary outcomes: condom-less anal sex, intentions to use condoms or condom self-efficacy, and HIV testing behaviors (Table 1). Outcomes will be assessed at all time points, allowing for initial differences to be compared as well as the longevity of these differences over the 9 months following the second intervention completion (ie, finishing SMART Squad or SSE2.0). Overall, 13 hypotheses were suggested within this primary comparison of SMART Squad versus SSE2.0 (Table 2).

**Table 2:** Power analysis by hypothesized group.

Hypothesis	Groups	Full (N)	80% reduction of full N after SSE^a^ due to response	Alpha values^b^	Power	Cohen effect size
1	All	1632	1306	.00207	0.999	0.52
2	Native American/Alaskan Native	200	160	.0083	0.830	0.52
3	Asian	200	160	.0083	0.830	0.52
4	Black	300	240	.00417	0.920	0.52
5	Latinx (English-speaking)	182	146	.00417	0.920	0.52
6	White	300	240	.00417	0.920	0.52
7	Native Hawaiian/Other Pacific Islanders	200	160	.0083	0.830	0.52
8	Latinx (Spanish-speaking)	250	200	.00102	0.939	0.52
9	Urban (nonrural)	1224	979	.00207	0.999	0.52
10	Rural	408	326	.00207	0.960	0.52
11	Low SES^c^	408	326	.00207	0.960	0.52
12	Mid/high SES	1224	979	.00207	0.999	0.52
13	Age	1632	1306	.00207	0.999	0.52

^a^SSE: SMART Sex Ed.

^b^The total alpha after Bonferroni adjustment was .05. The full sample size will be increased by 15% to account for projected attrition to a final total of 1878. The 15% increase will be equally distributed across all subgroups (ie, Native American/Alaskan Native, Asian, Black, Latinx-English, White, Native Hawaiian/Other Pacific Islander, and Latinx-Spanish).

^c^SES: socioeconomic status

Specifically, we will test the hypothesis (H1) of no difference in actual condom use or intentions/self-efficacy to use condoms in the SMART Squad group relative to the control group (SSE2.0; Figure 1, letter A). To understand the potential effects of the interventions on health disparities, we will test the hypothesis of no difference in actual condom use or intentions/self-efficacy to use condoms in the SMART Squad group relative to the control group separately within each of the 6 National Institutes of Health (NIH)–defined racial and ethnic categories (Table 2, H2-H7). Furthermore, we will test this hypothesis for the SMART Squad group relative to the control group among subjects: residing in nonrural areas (H9), residing in rural areas (H10), identified as low socioeconomic status (SES) according to a family affluence scale (H11), identified as medium or high SES according to a family affluence scale (H12), and with younger and older ages (H13). As we offer SMART and its interventions in Spanish, we will also test the effectiveness of SMART Squad (in Spanish) specifically among Spanish speakers (H8) relative to the control group (SSE2.0 in Spanish).

We will test each of these hypotheses using a 2-sided difference of proportions *t* test. For age, we seek to enroll approximately equal numbers of each age, and we will test for a significant interaction between treatment (SMART Squad vs SSE2.0) and age using a logistic regression model. We will use a Bonferroni multiplicity adjustment to ensure that the family-wise error rate of testing H1 to H13 is no greater than 0.05. Power calculations displayed in Table 2 show that even after this multiplicity adjustment, there is sufficient power to detect a moderate difference (ie, a Cohen effect size of 0.52) [107] in the proportion of responders with 80% power at the proposed sample size within each subgroup considered. All power calculations were performed using the *pwr* package in the R programming language. Table 2 shows that we have apportioned the Type I error inversely with the anticipated size of each subgroup, thereby ensuring sufficient power in the smaller subgroups. Finally, to account for attrition, we inflate each group’s sample size shown in Table 2 by 15% for a total proposed sample size of 1878.

We will also conduct a series of exploratory (ie, hypothesis generating) comparisons between interventions applied to nonresponders to SSE and SSE2.0. First, we will compare the response rates at 9 months among those assigned to SMART Squad with those assigned to SMART Sessions. This may provide evidence about whether the more intensive and costly SMART Sessions are more effective than SMART Squad among those that did not respond to the control condition/SSE2.0 (Figure 1, letter B). Second, we will compare response rates at 9 months among nonresponders to SMART Squad assigned to SMART Squad Booster 2 relative to those assigned to SMART Sessions. This will provide evidence about whether those who do not respond to SMART Squad will benefit from SMART Sessions or whether continued access to SMART Squad content would be sufficient (Figure 1, letter C). Finally, among responders to SSE, we will compare response rates at 9 months among those assigned to SMART Squad relative to follow-up only. This will provide evidence about whether those that respond to information only, web-based HIV education intervention (eg, SSE), will see additional benefits from SMART Squad (Figure 1, letter D). Unlike the primary comparisons, secondary analyses will not involve statistical tests of significance, but rather will consist of descriptive statistics, visualizations, and (unadjusted for multiplicity) confidence intervals. These results will be reported as exploratory.

In addition to these preceding exploratory hypotheses, we will use the data collected in this trial to estimate optimal individualized treatment strategies. An individualized treatment strategy is a sequence of decision rules, one per stage of intervention, which maps up-to-date patient information to a recommended intervention [108-110]. An optimal individualized strategy maximizes the total response rate by compounding the interventions’ effects (eg, SSE with SMART Squad or no SSE with SMART Squad and SMART Sessions), resulting in the best outcome for a potential user. A primary advantage of sequential multiple assignment designs is that they facilitate the estimation of an optimal individualized strategy. We will apply Q-learning [108,111,112] to estimate an optimal individualized strategy. To ensure that the strategy is interpretable given easily measurable data (eg, sexual activity, age) and thereby maximally informative for subsequent research, we will estimate an individualized strategy composed of decision rules represented as a sequence of if-then clauses [113]. For example, decision rules might be: if a subject is 16 years of age or older and has not experienced anal sex yet, assign them to SSE followed by SSE2.0, otherwise assign them to SMART Squad.

Our hybrid type 1 trial will also analyze data collected around the implementation of SMART. Guided by the RE-AIM framework [56,58,104], we will describe our ability to reach diverse AMSM through our recruitment efforts during the trial. We will also interview potential future implementers (ie, community-based organizations, CBOs) to understand what implementation strategies they might need to reach this population. AMSM ratings of acceptability and engagement with SMART (eg, completion rates, time through interventions, SMART coach satisfaction, and qualitative feedback) will supplement the primary efficacy outcomes as well as inform updates and improvements to the intervention over time. Determinants of adoption will be examined primarily through interviews and surveys with AMSM and CBOs to identify, respectively, actual and potential barriers and facilitators of uptake, drawing on the CFIR for key constructs [59]. We will assess the implementation needs of SMART by tracking workflow, operations, and other process metrics. Finally, to inform maintenance, we will assess the potential cost savings associated with implementing SMART using an HIV mathematical model that factors in the construction and delivery of the program, costs of future medical care, HIV incidence projections, quality of life weights, and other necessary inputs [114]. The model will estimate the 5-year and 10-year flow of fund differences for example individuals, Medicaid, private insurers, and other payers under specific assumptions, as well as cost utility estimates.

## Results

Between April 2018 and June 2020, 1285 AMSM had completed all baseline assessment components and were considered enrolled in the study. Of those enrolled, 357 AMSM have completed their 12-month follow-up survey and have finished participating in SMART. We proposed enrollment of 1878 AMSM, with recruitment concluding at the end of June 2020. The final sample will be diverse in terms of race and ethnicity, primary language spoken (ie, English and Spanish), geographic region, socioeconomic status, and urban versus rural location.

## Discussion

### Principal Findings

This hybrid type 1 evaluation of SMART, a promising stepped-care eHealth HIV prevention intervention for AMSM, is an important contribution to the field of HIV prevention and implementation science for several reasons. It also represents the first HIV prevention intervention to overcome linguistic barriers and target monolingual, Spanish-speaking adolescents. To begin, SMART delivers sexual health education on the web and directs to AMSM, circumventing many of the individual and structural barriers of traditional in-person curricula. SSE and SMART Squad, the first two intervention tiers, are available on any smartphone or internet-ready device at any time of day, and can be completed at the participants’ own paces. SMART Sessions are available via Skype and FaceTime, 2 readily used video chat platforms among teens, and allow participants to set up their sessions on their own terms and schedules. This level of availability and usability also helps reduce fears about being outed by the intervention itself.

Second, SMART provides a tailored curriculum for AMSM that addresses topics and concerns that are more prevalent among sexual minorities. SSE was modeled from a previously developed and tested intervention for AMSM [52], covering topics such as HIV risk differences between receptive and insertive anal sex, using a receptive or internal condom, water-based lubrications and their use during anal sex, how to come out to parents, and how to find support as a sexual minority. SSE and SMART Squad were developed with continuous input from a web-based youth advisory council of racially and ethnically diverse 13- to 18-year olds. Several members of that same youth advisory council read and helped revise the 120-page soap opera script for SMART Squad. Both interventions were beta-tested with AMSM. Sessions were pilot-tested before the randomized trial with 13- to 18-year olds and workshopped according to feedback from pilot coaches.

Third, SMART is the first trial testing the IMB model with AMSM and using intervention responsiveness as a benchmark before providing additional content or treatment to participants. Because interventions can be costly and potentially unnecessary if participants are already enacting change [115], it is necessary to find the right dose for AMSM regarding HIV information, situational and contextual behavioral motivations, and prevention skills. Sequentially designed programs that increase in intensity, such as SMART, may be the best way to maximize positive behavioral health change while minimizing overall cost [116]. They may also be an excellent means to identify moderating individual conditions that make some more likely to need increased prevention education (eg, if AMSM come from school districts that teach abstinence-only sexual education).

Finally, our use of a hybrid type 1 design will be the first-time implementation science data that will be prioritized during the creation and testing of an HIV intervention for AMSM. The NIH has invested heavily in developing eHealth HIV prevention programs; however, few to date have seen widespread use and none have targeted AMSM. This formative work helps us identify appropriate and feasible implementation strategies needed in the future to deploy SMART in the real world. Implementation data allow us to explore contextual determinants (ie, barriers and facilitators) to future dissemination, as well as preliminary implementation outcomes. It also indicates how we might update the content and technology of SMART over time to avoid obsolescence. More broadly, the data collected on SMART’s reach, engagement, cost, adoption, and maintenance will be invaluable for future researchers as they create web-based and in-person sexual health curricula. It can also provide insight and direction for CBOs and other institutions (eg, schools) that may be interested in upgrading their prevention programs to a web-based platform and to target AMSM.

### Limitations

There are several limitations that SMART faces in its current form while we actively enroll AMSM. SMART is an eHealth intervention, which means that for SSE, SMART Squad, and SSE2.0, study staff are not present when participants access and move through intervention content. If participants have questions or concerns while viewing materials, there is no synchronously available moderator to help. Similarly, if participants encounter technical problems while viewing any content, the onus is on the participant to contact the study staff and report the issue. To counteract these potential issues, we include feedback pages across all the interventions, at multiple points within modules, to elicit questions, concerns, and participant attitudes. Open-ended textboxes are available, along with clickable rating buttons. We also have an active process to catalog the feedback, change content when appropriate, and respond to participants. Similarly, if a participant encounters a technical issue, the SMART toolbar has a dedicated button called *Technical Help*, which allows participants within the intervention to send study staff reports of the issue. The SMART platform automatically codes the message with the participant’s browser, device, platform, and device operating system version.

Participant attention during the intervention is another potential concern. During SSE, SMART Squad, and SSE2.0, how intently participants are focusing on the content cannot be measured precisely. Given other web-based (eg, social media and television) and offline distractions (eg, homework, chores, and extracurricular activities), it may be possible that participants are focusing less on SMART content than if delivered in person using a traditional modality such as lectures or discussions. We do measure time-through-intervention; although few participants appear to rush through the intervention (eg, viewing for 10 min or less), overall focus may be inconsistent and an unmeasured individual participant difference. During SMART Sessions, SMART coaches have anecdotally indicated several cases in which they suspected participants were multitasking using other apps while engaging in discussions. In these cases, coaches acknowledge that the participant may be distracted and attempt to refocus the individual or reschedule the session.

In addition to these operational limitations, there is a larger issue of trying to test a SMART intervention with such a young population. This type of trial requires participants to engage with multiple interventions of varying intensities and lengths. More than 90% of the participants will ultimately receive at least 2 interventions, if not 3, over the course of 12 months. Considering that many of these participants might not be intrinsically motivated or interested in sexual health education, this amount of content may exceed participant interest. Granted, months transpire between interventions; this remains to be a potential problem when working with adolescents who already are saturated with formal and informal education on a daily basis.

### Conclusions

Despite these limitations, the randomized trial of SMART currently shows that eHealth, stepped-care sequenced interventions are implementable for AMSM. The trial is planned to finish in the fourth quarter of 2021. Providing sexual education to AMSM, an underserved population for HIV prevention interventions, recognizes the importance of attending to their unique needs if we will end the domestic HIV epidemic [117]. Reducing the number of HIV infections for this youngest at-risk population dramatically reduces lifetime HIV costs and decreases the overall number of HIV quality-adjusted life years [118]. Most importantly, programs such as SMART may ultimately prevent or delay HIV infection among AMSM. Considering this is a population that consistently fails to test for HIV and inconsistently uses HIV protective measures, preventing infections for AMSM is a high-priority public health activity.

## Appendix

Multimedia Appendix 1 CONSORT-EHEALTH checklist (V. 1. 6. 1).

## Abbreviations

AMSM: adolescent men who have sex with men
CBO: community-based organization
CDC: Centers for Disease Control and Prevention
CFIR: Consolidated Framework for Implementation Research
G2G: Guy2Guy
IMB: information-motivation-behavioral skills
LGBTQ: lesbian, gay, bisexual, transgender, queer
MI: motivational interviewing
MITI: motivational interviewing treatment integrity
MSM: men who have sex with men
NIH: National Institutes of Health
PrEP: pre-exposure prophylaxis
QSE: Queer Sex Ed
RE-AIM: Reach, Effectiveness, Adoption, Implementation, and Maintenance
REDCap: Research Electronic Data Capture
SES: socioeconomic status
SSE: SMART Sex Ed
SSE2.0: SMART Sex Ed 2.0
STI: sexually transmitted infection
