# Supplementary material for: Evaluation of a Stepped-Care eHealth HIV Prevention Program for Diverse Adolescent Men Who Have Sex With Men: Protocol for a Hybrid Type 1 Effectiveness Implementation Trial of SMART
Source: JMIR Res Protoc. 2020 Aug 11;9(8):e19701. doi: 10.2196/19701 (PMC7448177; doi:10.2196/19701)
Supplement: Multimedia Appendix 1 [file resprot_v9i8e19701_app1.pdf]

CONSORT-EHEALTH (V 1.6.1) - Submission/Publication Form

The CONSORT-EHEALTH checklist is intended for authors of randomized trials evaluating web-based and Internet-based applications/interventions, including mobile interventions, electronic games (incl multiplayer games), social media, certain telehealth applications, and other interactive and/or networked electronic applications. Some of the items (e.g. all subitems under item 5 - description of the intervention) may also be applicable for other study designs.

The goal of the CONSORT EHEALTH checklist and guideline is to be
a) a guide for reporting for authors of RCTs,
b) to form a basis for appraisal of an ehealth trial (in terms of validity)

CONSORT-EHEALTH items/subitems are MANDATORY reporting items for studies published in the Journal of Medical Internet Research and other journals / scientific societies endorsing the checklist.

Items numbered 1., 2., 3., 4a., 4b etc are original CONSORT or CONSORT-NPT (non-pharmacologic treatment) items.
Items with Roman numerals (i., ii, iii, iv etc.) are CONSORT-EHEALTH extensions/clarifications.

As the CONSORT-EHEALTH checklist is still considered in a formative stage, we would ask that you also RATE ON A SCALE OF 1-5 how important/useful you feel each item is FOR THE PURPOSE OF THE CHECKLIST and reporting guideline (optional).

Mandatory reporting items are marked with a red *.
In the textboxes, either copy & paste the relevant sections from your manuscript into this form - please include any quotes from your manuscript in QUOTATION MARKS,
or answer directly by providing additional information not in the manuscript, or elaborating on why the item was not relevant for this study.

YOUR ANSWERS WILL BE PUBLISHED AS A SUPPLEMENTARY FILE TO YOUR PUBLICATION IN JMIR AND ARE CONSIDERED PART OF YOUR PUBLICATION (IF ACCEPTED).
Please fill in these questions diligently. Information will not be copyedited, so please use proper spelling and grammar, use correct capitalization, and avoid abbreviations.

DO NOT FORGET TO SAVE AS PDF _AND_ CLICK THE SUBMIT BUTTON SO YOUR ANSWERS ARE IN OUR DATABASE !!!

Citation Suggestion (if you append the pdf as Appendix we suggest to cite this paper in the caption):
Eysenbach G, CONSORT-EHEALTH Group
CONSORT-EHEALTH: Improving and Standardizing Evaluation Reports of Web-based and

Mobile Health Interventions
J Med Internet Res 2011;13(4):e126
URL: http://www.jmir.org/2011/4/e126/
doi: 10.2196/jmir.1923
PMID: 22209829

* Required

Your name *

First Last

David Moskowitz

Primary Affiliation (short), City, Country *

University of Toronto, Toronto, Canada

Northwestern University, Feinberg School of M

Your e-mail address *

abc@gmail.com

david.moskowitz@northwestern.edu

Title of your manuscript *

Provide the (draft) title of your manuscript.

Evaluation of a stepped care eHealth HIV prevention program for diverse adolescent MSM: Protocol for a hybrid type 1 effectiveness-implementation trial of SMART

Name of your App/Software/Intervention *

If there is a short and a long/alternate name, write the short name first and add the long name in brackets.

SMART (SMART Program)

Evaluated Version (if any)

e.g. "V1", "Release 2017-03-01", "Version 2.0.27913"

Your answer

Language(s) *

What language is the intervention/app in? If multiple languages are available, separate by comma (e.g. "English, French")

English

URL of your Intervention Website or App

e.g. a direct link to the mobile app on app in appstore (itunes, Google Play), or URL of the website. If the intervention is a DVD or hardware, you can also link to an Amazon page.

https://smart.northwestern.edu/

URL of an image/screenshot (optional)

Your answer

Accessibility *

Can an enduser access the intervention presently?

- ○ access is free and open
- ◉ access only for special usergroups, not open
- ○ access is open to everyone, but requires payment/subscription/in-app purchases
- ○ app/intervention no longer accessible
- ○ Other:

Primary Medical Indication/Disease/Condition *

e.g. "Stress", "Diabetes", or define the target group in brackets after the condition, e.g. "Autism (Parents of children with)", "Alzheimers (Informal Caregivers of)"

HIV (HIV Prevention for adolescent men who h

Primary Outcomes measured in trial *

comma-separated list of primary outcomes reported in the trial

Number of condomless anal sex partners, con

Secondary/other outcomes

Are there any other outcomes the intervention is expected to affect?

HIV knowledge, HIV motivation and behavioral skills, condom errors

Recommended "Dose" *

What do the instructions for users say on how often the app should be used?

- ○ Approximately Daily
- ○ Approximately Weekly
- ○ Approximately Monthly
- ○ Approximately Yearly
- ◉ "as needed"
- ○ Other:

Approx. Percentage of Users (starters) still using the app as recommended after 3 months *

- ◉ unknown / not evaluated
- ○ 0-10%
- ○ 11-20%
- ○ 21-30%
- ○ 31-40%
- ○ 41-50%
- ○ 51-60%
- ○ 61-70%
- ○ 71%-80%
- ○ 81-90%
- ○ 91-100%
- ○ Other:

Overall, was the app/intervention effective? *

- ( ) yes: all primary outcomes were significantly better in intervention group vs control
- ( ) partly: SOME primary outcomes were significantly better in intervention group vs control
- ( ) no statistically significant difference between control and intervention
- ( ) potentially harmful: control was significantly better than intervention in one or more outcomes
- ( ) inconclusive: more research is needed
- (•) Other: app/intervention effectiveness has not yet been assessed; more follo

Article Preparation Status/Stage *

At which stage in your article preparation are you currently (at the time you fill in this form)

- ( ) not submitted yet - in early draft status
- ( ) not submitted yet - in late draft status, just before submission
- ( ) submitted to a journal but not reviewed yet
- (•) submitted to a journal and after receiving initial reviewer comments
- ( ) submitted to a journal and accepted, but not published yet
- ( ) published
- ( ) Other:

Journal *

If you already know where you will submit this paper (or if it is already submitted), please provide the journal name (if it is not JMIR, provide the journal name under "other")

- ○ not submitted yet / unclear where I will submit this
- ○ Journal of Medical Internet Research (JMIR)
- ○ JMIR mHealth and UHealth
- ○ JMIR Serious Games
- ○ JMIR Mental Health
- ○ JMIR Public Health
- ○ JMIR Formative Research
- ◉ Other JMIR sister journal
- ○ Other:

Is this a full powered effectiveness trial or a pilot/feasibility trial? *

- ○ Pilot/feasibility
- ◉ Fully powered

Manuscript tracking number *

If this is a JMIR submission, please provide the manuscript tracking number under "other" (The ms tracking number can be found in the submission acknowledgement email, or when you login as author in JMIR. If the paper is already published in JMIR, then the ms tracking number is the four-digit number at the end of the DOI, to be found at the bottom of each published article in JMIR)

- ○ no ms number (yet) / not (yet) submitted to / published in JMIR
- ◉ Other: JMIR ms#18750

TITLE AND ABSTRACT

1a) TITLE: Identification as a randomized trial in the title

1a) Does your paper address CONSORT item 1a? *

I.e does the title contain the phrase "Randomized Controlled Trial"? (if not, explain the reason under "other")

- (selected) yes
- ( ) Other:

1a-i) Identify the mode of delivery in the title

Identify the mode of delivery. Preferably use “web-based” and/or “mobile” and/or “electronic game” in the title. Avoid ambiguous terms like “online”, “virtual”, “interactive”. Use “Internet-based” only if Intervention includes non-web-based Internet components (e.g. email), use “computer-based” or “electronic” only if offline products are used. Use “virtual” only in the context of “virtual reality” (3-D worlds). Use “online” only in the context of “online support groups”. Complement or substitute product names with broader terms for the class of products (such as “mobile” or “smart phone” instead of “iphone”), especially if the application runs on different platforms.

	1	2	3	4	5	
subitem not at all important	○	●	○	○	○	essential

Clear selection

Does your paper address subitem 1a-i? *

Copy and paste relevant sections from manuscript title (include quotes in quotation marks "like this" to indicate direct quotes from your manuscript), or elaborate on this item by providing additional information not in the ms, or briefly explain why the item is not applicable/relevant for your study

stepped care "eHealth" HIV prevention program. (Rationale: Because the intervention uses mixed-mediums of web-based and mobile-based platforms, eHealth was the most comprehensive descriptor).

1a-ii) Non-web-based components or important co-interventions in title

Mention non-web-based components or important co-interventions in title, if any (e.g., “with telephone support”).

	1	2	3	4	5	
subitem not at all important	(•)	( )	( )	( )	( )	essential

Clear selection

Does your paper address subitem 1a-ii?

Copy and paste relevant sections from manuscript title (include quotes in quotation marks "like this" to indicate direct quotes from your manuscript), or elaborate on this item by providing additional information not in the ms, or briefly explain why the item is not applicable/relevant for your study

This is an exclusively online/mobile intervention with no non-web-based components.

1a-iii) Primary condition or target group in the title

Mention primary condition or target group in the title, if any (e.g., “for children with Type I Diabetes”) Example: A Web-based and Mobile Intervention with Telephone Support for Children with Type I Diabetes: Randomized Controlled Trial

	1	2	3	4	5	
subitem not at all important	( )	( )	( )	( )	(•)	essential

Clear selection

Does your paper address subitem 1a-iii? *

Copy and paste relevant sections from manuscript title (include quotes in quotation marks "like this" to indicate direct quotes from your manuscript), or elaborate on this item by providing additional information not in the ms, or briefly explain why the item is not applicable/relevant for your study

"diverse adolescent MSM"

1b) ABSTRACT: Structured summary of trial design, methods, results, and conclusions

NPT extension: Description of experimental treatment, comparator, care providers, centers, and blinding status.

1b-i) Key features/functionalities/components of the intervention and comparator in the METHODS section of the ABSTRACT

Mention key features/functionalities/components of the intervention and comparator in the abstract. If possible, also mention theories and principles used for designing the site. Keep in mind the needs of systematic reviewers and indexers by including important synonyms. (Note: Only report in the abstract what the main paper is reporting. If this information is missing from the main body of text, consider adding it)

	1	2	3	4	5	
subitem not at all important	○	◉	○	○	○	essential

Clear selection

Does your paper address subitem 1b-i? *

Copy and paste relevant sections from the manuscript abstract (include quotes in quotation marks "like this" to indicate direct quotes from your manuscript), or elaborate on this item by providing additional information not in the ms, or briefly explain why the item is not applicable/relevant for your study

"Using a sequential multiple assignment randomized trial design, we will evaluate the impact of a stepped care package of increasingly intensive eHealth interventions (i.e., the universal, information-based SMART Sex Ed; the more intensive, selective SMART Squad; and a higher-cost, indicated SMART Sessions)."

1b-ii) Level of human involvement in the METHODS section of the ABSTRACT

Clarify the level of human involvement in the abstract, e.g., use phrases like “fully automated” vs. “therapist/nurse/care provider/physician-assisted” (mention number and expertise of providers involved, if any). (Note: Only report in the abstract what the main paper is reporting. If this information is missing from the main body of text, consider adding it)

	1	2	3	4	5	
subitem not at all important	○	◉	○	○	○	essential

Clear selection

Does your paper address subitem 1b-ii?

Copy and paste relevant sections from the manuscript abstract (include quotes in quotation marks "like this" to indicate direct quotes from your manuscript), or elaborate on this item by providing additional information not in the ms, or briefly explain why the item is not applicable/relevant for your study

"SMART is the first online program for AMSM to take a stepped-care approach to sexual education and HIV prevention. This design means SMART delivers resources to all adolescents, but more costly treatments (e.g., videochat counseling in SMART Sessions) are conserved for individuals who need them most."

1b-iii) Open vs. closed, web-based (self-assessment) vs. face-to-face assessments in the METHODS section of the ABSTRACT

Mention how participants were recruited (online vs. offline), e.g., from an open access website or from a clinic or a closed online user group (closed usergroup trial), and clarify if this was a purely web-based trial, or there were face-to-face components (as part of the intervention or for assessment). Clearly say if outcomes were self-assessed through questionnaires (as common in web-based trials). Note: In traditional offline trials, an open trial (open-label trial) is a type of clinical trial in which both the researchers and participants know which treatment is being administered. To avoid confusion, use “blinded” or “unblinded” to indicated the level of blinding instead of “open”, as “open” in web-based trials usually refers to “open access” (i.e. participants can self-enrol). (Note: Only report in the abstract what the main paper is reporting. If this information is missing from the main body of text, consider adding it)

	1	2	3	4	5	
subitem not at all important	○	◉	○	○	○	essential

Clear selection

Does your paper address subitem 1b-iii?

Copy and paste relevant sections from the manuscript abstract (include quotes in quotation marks "like this" to indicate direct quotes from your manuscript), or elaborate on this item by providing additional information not in the ms, or briefly explain why the item is not applicable/relevant for your study

"Participants are recruited primarily from social media sources, using paid and unpaid advertisements." "Assessments of intervention outcomes at 3-, 6-, 9-, and 12-months are ongoing."

1b-iv) RESULTS section in abstract must contain use data

Report number of participants enrolled/assessed in each group, the use/uptake of the intervention (e.g., attrition/adherence metrics, use over time, number of logins etc.), in addition to primary/secondary outcomes. (Note: Only report in the abstract what the main paper is reporting. If this information is missing from the main body of text, consider adding it)

	1	2	3	4	5	
subitem not at all important	○	○	◉	○	○	essential

Clear selection

Does your paper address subitem 1b-iv?

Copy and paste relevant sections from the manuscript abstract (include quotes in quotation marks "like this" to indicate direct quotes from your manuscript), or elaborate on this item by providing additional information not in the ms, or briefly explain why the item is not applicable/relevant for your study

To date, the trial has enrolled 1,285 AMSM ages 13-18, with a target enrollment of 1,878. Recruitment concluded in June 2020. Participants were recruited from 49 of the United States, as well as Puerto Rico, and the District of Columbia.

1b-v) CONCLUSIONS/DISCUSSION in abstract for negative trials

Conclusions/Discussions in abstract for negative trials: Discuss the primary outcome - if the trial is negative (primary outcome not changed), and the intervention was not used, discuss whether negative results are attributable to lack of uptake and discuss reasons. (Note: Only report in the abstract what the main paper is reporting. If this information is missing from the main body of text, consider adding it)

	1	2	3	4	5	
subitem not at all important	○	◉	○	○	○	essential

Clear selection

Does your paper address subitem 1b-v?

Copy and paste relevant sections from the manuscript abstract (include quotes in quotation marks "like this" to indicate direct quotes from your manuscript), or elaborate on this item by providing additional information not in the ms, or briefly explain why the item is not applicable/relevant for your study

SMART has the potential to reach AMSM to provide them with a sex-positive curriculum that empowers them with the information, motivation, and skills to make better health choices. (The trial is ongoing so the precise conclusions cannot be drawn at this time).

INTRODUCTION

2a) In INTRODUCTION: Scientific background and explanation of rationale

2a-i) Problem and the type of system/solution

Describe the problem and the type of system/solution that is object of the study: intended as stand-alone intervention vs. incorporated in broader health care program? Intended for a particular patient population? Goals of the intervention, e.g., being more cost-effective to other interventions, replace or complement other solutions? (Note: Details about the intervention are provided in “Methods” under 5)

	1	2	3	4	5	
subitem not at all important	○	○	○	○	◉	essential

Clear selection

Does your paper address subitem 2a-i? *

Copy and paste relevant sections from the manuscript (include quotes in quotation marks "like this" to indicate direct quotes from your manuscript), or elaborate on this item by providing additional information not in the ms, or briefly explain why the item is not applicable/relevant for your study

"Current evidence-based HIV prevention programs focus primarily on adults and heterosexual youth [3]. However, as the issues affecting sexual health decisions among AMSM are unique (e.g., access to affirming care) [4, 5], interventions should be designed with their needs in mind to ensure the content resonates with them. Moreover, prevention programs need to be responsive to racial and ethnic minority AMSM who experience reduced access to HIV/STI prevention services [6, 7], and as a corollary, increased HIV incidence [1]. eHealth interventions represent a critical modality for delivering AMSM-specific intervention material where youth “are,” considering 97% of adolescents across all races and income levels are online every day [8].

2a-ii) Scientific background, rationale: What is known about the (type of) system

Scientific background, rationale: What is known about the (type of) system that is the object of the study (be sure to discuss the use of similar systems for other conditions/diagnoses, if appropiate), motivation for the study, i.e. what are the reasons for and what is the context for this specific study, from which stakeholder viewpoint is the study performed, potential impact of findings [2]. Briefly justify the choice of the comparator.

	1	2	3	4	5	
subitem not at all important	○	○	○	◉	○	essential

Clear selection

Does your paper address subitem 2a-ii? *

Copy and paste relevant sections from the manuscript (include quotes in quotation marks "like this" to indicate direct quotes from your manuscript), or elaborate on this item by providing additional information not in the ms, or briefly explain why the item is not applicable/relevant for your study

"Combining online recruitment with intervention delivery across a range of devices could overcome many access barriers to engagement of AMSM in HIV prevention. Here, we describe a hybrid type 1 effectiveness-implementation protocol [9] aimed at testing the SMART Program's effectiveness and informing future implementation as a service. Our study uses a sequential multiple assignment randomized trial [10, 11] to examine the effectiveness of each component of SMART, which consists of three eHealth HIV prevention interventions."

2b) In INTRODUCTION: Specific objectives or hypotheses

Does your paper address CONSORT subitem 2b? *

Copy and paste relevant sections from the manuscript (include quotes in quotation marks "like this" to indicate direct quotes from your manuscript), or elaborate on this item by providing additional information not in the ms, or briefly explain why the item is not applicable/relevant for your study

"We are testing for individual intervention and cumulative intervention effectiveness at reducing condomless anal sex, and increasing condom use intentions, self-efficacy, and HIV testing among AMSM participants. Additionally, we are testing whether SMART has differential effectiveness across sub-groups of AMSM based on race/ethnicity, urban/rural residence, age, socioeconomic status, and preference for an English versus Spanish language version of the intervention. Our use of a hybrid type 1 design simultaneously allows us to collect data that will provide critical insight into factors that may impact SMART's real-world implementation."

METHODS

3a) Description of trial design (such as parallel, factorial) including allocation ratio

Does your paper address CONSORT subitem 3a? *

Copy and paste relevant sections from the manuscript (include quotes in quotation marks "like this" to indicate direct quotes from your manuscript), or elaborate on this item by providing additional information not in the ms, or briefly explain why the item is not applicable/relevant for your study

"This study uses a hybrid type 1, sequential multiple assignment randomized trial [10, 11, 63], evaluating the impact of a package of increasingly intensive, stepped care interventions (see Figure 1). The virtue of using a sequential design is that it can help determine which of a wide variety of intervention strategies (or combinations therein) will be best suited to a given individual, thus maximizing efficacy. Previous versions of SMART intervention steps have already shown evidence of efficacy with diverse young adult MSM [52, 64, 65] and in this study were further developmentally and linguistically adapted to accommodate the unique social experiences and health barriers of English- and Spanish-speaking adolescents [66]. All participants receive the universally relevant SSE intervention at baseline. Response to the intervention, as defined in the section below, will be measured at the 3-month follow-up assessment. Those who respond to SSE will be randomized to receive either SS or to a follow-up only condition. Those who do not respond to SSE will be randomized to receive one of four treatment packages, two of which include the control condition, SMART Sex Ed 2.0 (SSE2.0). Shown within Figure 1, these treatment packages represent pathways a participant could take through the trial contingent on their responder status, and thus are termed “embedded regimes” (ER)."

3b) Important changes to methods after trial commencement (such as eligibility criteria), with reasons

Does your paper address CONSORT subitem 3b? *

Copy and paste relevant sections from the manuscript (include quotes in quotation marks "like this" to indicate direct quotes from your manuscript), or elaborate on this item by providing additional information not in the ms, or briefly explain why the item is not applicable/relevant for your study

N/A; No changes have been made since the commencement of the trial

3b-i) Bug fixes, Downtimes, Content Changes

Bug fixes, Downtimes, Content Changes: ehealth systems are often dynamic systems. A description of changes to methods therefore also includes important changes made on the intervention or comparator during the trial (e.g., major bug fixes or changes in the functionality or content) (5-iii) and other “unexpected events” that may have influenced study design such as staff changes, system failures/downtimes, etc. [2].

	1	2	3	4	5	
subitem not at all important	○	○	○	◉	○	essential

Clear selection

Does your paper address subitem 3b-i?

Copy and paste relevant sections from the manuscript (include quotes in quotation marks "like this" to indicate direct quotes from your manuscript), or elaborate on this item by providing additional information not in the ms, or briefly explain why the item is not applicable/relevant for your study

N/A; No major bugs or problems have impacted or halted the trial

4a) Eligibility criteria for participants

Does your paper address CONSORT subitem 4a? *

Copy and paste relevant sections from the manuscript (include quotes in quotation marks "like this" to indicate direct quotes from your manuscript), or elaborate on this item by providing additional information not in the ms, or briefly explain why the item is not applicable/relevant for your study

"Potential participants are eligible for this study according to the following inclusion criteria: 1) they were assigned male at birth; 2) they identify as a sexual minority (i.e., report their sexual orientation as gay, bisexual, queer, lesbian, or pansexual) or report attraction to cisgender males; 3) they report an HIV-negative or unknown HIV status; 4) they have engaged in sexual contact with another person (defined as having touched another person's genitals, or performed oral, vaginal, or anal sex); 5) are between the ages of 13 and 18 years old (inclusive); 6) have access to/use the internet; 7) are able to read and speak English or Spanish at an 6th grade level or better; 8) and reside in the United States, including Puerto Rico, Guam, and the U.S. Virgin Islands. Current gender identity was not an inclusion/exclusion criteria. Those assigned male at birth could identify as any gender identity (e.g., transgender, nonbinary, genderqueer, genderfluid) provided they met the eight inclusion criteria. Those identifying as intersex or assigned female at birth were excluded to comply with the trail's specific aim to curtail HIV spread in AMSM."

4a-i) Computer / Internet literacy

Computer / Internet literacy is often an implicit "de facto" eligibility criterion - this should be explicitly clarified.

	1	2	3	4	5	
subitem not at all important	○	○	○	◉	○	essential

Clear selection

Does your paper address subitem 4a-i?

Copy and paste relevant sections from the manuscript (include quotes in quotation marks "like this" to indicate direct quotes from your manuscript), or elaborate on this item by providing additional information not in the ms, or briefly explain why the item is not applicable/relevant for your study

"...6) have access to/use the internet; 7) are able to read and speak English or Spanish at an 6th grade level or better..."

4a-ii) Open vs. closed, web-based vs. face-to-face assessments:

Open vs. closed, web-based vs. face-to-face assessments: Mention how participants were recruited (online vs. offline), e.g., from an open access website or from a clinic, and clarify if this was a purely web-based trial, or there were face-to-face components (as part of the intervention or for assessment), i.e., to what degree got the study team to know the participant. In online-only trials, clarify if participants were quasi-anonymous and whether having multiple identities was possible or whether technical or logistical measures (e.g., cookies, email confirmation, phone calls) were used to detect/prevent these.

	1	2	3	4	5	
subitem not at all important	○	○	○	◉	○	essential

Clear selection

Does your paper address subitem 4a-ii? *

Copy and paste relevant sections from the manuscript (include quotes in quotation marks "like this" to indicate direct quotes from your manuscript), or elaborate on this item by providing additional information not in the ms, or briefly explain why the item is not applicable/relevant for your study

“English- and Spanish-speaking AMSM are recruited using paid advertising on social media (e.g., Instagram, Facebook), and active online engagement through geospatial dating applications and other social media outlets (e.g., reddit, tumblr). Advertisements, posts, and direct messages send potential participants to a brief online eligibility survey, available in English and Spanish. Participants who complete it in English are given access to the English-only version of SMART. Those who complete it in Spanish are given access to the Spanish-only version of SMART, in which all study consent, communications/reminders, intervention content, and assessments are provided in Spanish. Figure 2 displays participant flow from advertisement to enrollment. All study surveys are administered via REDCap [71]. The Northwestern University IRB granted SMART a waiver of signed documentation of informed consent/assent, as well as a waiver of parental permission for participants under 18. Participants are routed to a consent page with four decisional capacity questions, which assess their comprehension of study tasks, risks, and benefits, as well as how to exit the study [72]. They also submit a username for study staff approval on the consent page. Usernames cannot have any personally identifying information (e.g., name, email). If they provide consent, study staff email and/or text prospective participants to set up a videochat to verify participant identity, review the study tasks, and answer any of their questions. During this 5-minute videochat, AMSM are also asked to explain back to study staff what they will be asked to do as a SMART participant. Finally, if a participant has submitted a username with personally identifying information, the study staff will work with the participant to revise the username while on the videochat.

Once the videochat is complete, participants are sent their baseline assessment survey, which has all pertinent primary and secondary study measures. Completion of the baseline assessment triggers an automatic email inviting the participant to login to SMART by going to the website, resetting their password using their username, and then logging in to access the first tier of the intervention. All participants, regardless of demographic characteristics or responses to their baseline assessment, are given access to SSE.”

4a-iii) Information giving during recruitment

Information given during recruitment. Specify how participants were briefed for recruitment and in the informed consent procedures (e.g., publish the informed consent documentation as appendix, see also item X26), as this information may have an effect on user self-selection, user expectation and may also bias results.

	1	2	3	4	5	
subitem not at all important	○	○	○	●	○	essential

Clear selection

Does your paper address subitem 4a-iii?

Copy and paste relevant sections from the manuscript (include quotes in quotation marks "like this" to indicate direct quotes from your manuscript), or elaborate on this item by providing additional information not in the ms, or briefly explain why the item is not applicable/relevant for your study

"If they provide consent, study staff email and/or text prospective participants to set up a videochat to verify participant identity, review the study tasks, and answer any of their questions. During this 5-minute videochat, AMSM are also asked to explain back to study staff what they will be asked to do as a SMART participant."

4b) Settings and locations where the data were collected

Does your paper address CONSORT subitem 4b? *

Copy and paste relevant sections from the manuscript (include quotes in quotation marks "like this" to indicate direct quotes from your manuscript), or elaborate on this item by providing additional information not in the ms, or briefly explain why the item is not applicable/relevant for your study

"participants are sent their online baseline assessment survey, which has all pertinent primary and secondary study measures."

4b-i) Report if outcomes were (self-)assessed through online questionnaires

Clearly report if outcomes were (self-)assessed through online questionnaires (as common in web-based trials) or otherwise.

	1	2	3	4	5	
subitem not at all important	○	○	◉	○	○	essential

Clear selection

Does your paper address subitem 4b-i? *

Copy and paste relevant sections from the manuscript (include quotes in quotation marks "like this" to indicate direct quotes from your manuscript), or elaborate on this item by providing additional information not in the ms, or briefly explain why the item is not applicable/relevant for your study

"participants are sent their online baseline assessment survey, which has all pertinent primary and secondary study measures."

4b-ii) Report how institutional affiliations are displayed

Report how institutional affiliations are displayed to potential participants [on ehealth media], as affiliations with prestigious hospitals or universities may affect volunteer rates, use, and reactions with regards to an intervention.(Not a required item – describe only if this may bias results)

	1	2	3	4	5	
subitem not at all important	◉	○	○	○	○	essential

Clear selection

Does your paper address subitem 4b-ii?

Copy and paste relevant sections from the manuscript (include quotes in quotation marks "like this" to indicate direct quotes from your manuscript), or elaborate on this item by providing additional information not in the ms, or briefly explain why the item is not applicable/relevant for your study

N/a

5) The interventions for each group with sufficient details to allow replication, including how and when they were actually administered

5-i) Mention names, credential, affiliations of the developers, sponsors, and owners

Mention names, credential, affiliations of the developers, sponsors, and owners [6] (if authors/evaluators are owners or developer of the software, this needs to be declared in a “Conflict of interest” section or mentioned elsewhere in the manuscript).

	1	2	3	4	5	
subitem not at all important	◉	○	○	○	○	essential

Clear selection

Does your paper address subitem 5-i?

Copy and paste relevant sections from the manuscript (include quotes in quotation marks "like this" to indicate direct quotes from your manuscript), or elaborate on this item by providing additional information not in the ms, or briefly explain why the item is not applicable/relevant for your study

N/A

5-ii) Describe the history/development process

Describe the history/development process of the application and previous formative evaluations (e.g., focus groups, usability testing), as these will have an impact on adoption/use rates and help with interpreting results.

	1	2	3	4	5	
subitem not at all important	○	○	◉	○	○	essential

Clear selection

Does your paper address subitem 5-ii?

Copy and paste relevant sections from the manuscript (include quotes in quotation marks "like this" to indicate direct quotes from your manuscript), or elaborate on this item by providing additional information not in the ms, or briefly explain why the item is not applicable/relevant for your study

"SSE represents the first-tier intervention for SMART. It is exclusively informational in nature and was adapted from an intervention previously tested on LGBTQ youth showing preliminary efficacy (i.e., Queer Sex Ed) [52]. As part of the adaptation process [90], core sexual health competencies and learning objectives from the CDC [91] and Sexuality Information and Education Council of the United States (SIECUS) [92] were incorporated, and if necessary, were updated to suit a sexual minority audience (e.g., coming out strategies). We assembled a diverse, standing online youth advisory council of AMSM (13-18 year olds) to review our adapted content and to answer questions about the relevance of information we were considering incorporating. Members of the council acted as an asynchronous focus group and were compensated monthly for their time [93]. Besides ensuring SSE content would resonate with AMSM, this focus group also allowed for community member stakeholders (i.e., AMSM) to participate in the intervention creation. SSE contains four modules that participants can navigate in any order they choose (see Figure 3). Media assets used across the modules include: full-page scroll screens (resembling social media feeds), slideshows with narration recorded using near-peer voice actors, videos, games, quizzes, and GIFs. Emojis are liberally used to make topics and lessons more tangible to participants, who commonly use emojis in online communication, including about sexual behavior. “SMART Facts” are used to segue between modules. They describe LGBTQ historical moments (e.g., the Stonewall riots) and LGBTQ-racial/ethnic identity intersectionality (e.g., pictures and a historic description of the Native Hawaiian LGBTQ experience). All modules end with a content quiz for participants, which helps them identify areas they may want to review. When participants select an incorrect response, they are given messaging that explains why their choice is incorrect and why another answer may be the better option." "SS represents the experimental second tier-intervention for SMART. Differing in many ways from SSE, SS focuses on improving participants’ motivations to concentrate on their sexual health and behavioral skills to enact protective measures to prevent HIV or STIs. This intervention was adapted from Keep it Up!, a CDC best-evidence effective intervention previously tested on young adult MSM [64, 94], using Intervention Mapping as a systematic approach [66]. Virtually all of the adapted content, including all scripted videos, were reviewed by our online youth advisory council. SS contains 6-episodes and 2-booster episodes; the first booster is delivered one-month following the completion of episode six and the second is delivered three-[illegible]

months following the completion of episode 6. Participants are forced to break for 8-hours between episodes 3 and 4. Figure 4 describes the main concepts and active learning components within each episode." "Sessions represents the third tier intervention for SMART. Similar to SS, this intervention focuses on motivations, skill building, and goal setting for participants. However, where SS uses an automated online platform, Sessions relies on one-on-one video-chat motivational interviewing counseling between participants and SMART Coaches. This program was developmentally adapted from Young Men's Health Project, an effective intervention previously tested on young adult MSM [65]. Sessions is delivered by clinical professionals with post-graduate training in counseling or psychology. All coaches receive extensive training in Motivational Interviewing (MI) techniques and conduct mock sessions in order to be cleared to deliver the intervention to SMART participants [95]. Coaches receive weekly individual and group supervision by a licensed clinical psychologist to ensure quality delivery of MI principles. Participants who are randomized to Sessions participate in 3-4 video chat sessions over the course of 4-6 weeks via Skype or FaceTime. The number of sessions is determined by the coach, based on whether the participant reports engagement in condomless sex and/or is a strong candidate for PrEP. Video chats last between 20-45 minutes, on average, and participants remain with the same coach for all of their sessions. Fifteen minutes is the minimum for being considered a completed session."

5-iii) Revisions and updating

Revisions and updating. Clearly mention the date and/or version number of the application/intervention (and comparator, if applicable) evaluated, or describe whether the intervention underwent major changes during the evaluation process, or whether the development and/or content was "frozen" during the trial. Describe dynamic components such as news feeds or changing content which may have an impact on the replicability of the intervention (for unexpected events see item 3b).

	1	2	3	4	5	
subitem not at all important	○	◉	○	○	○	essential

Clear selection

Does your paper address subitem 5-iii?

Copy and paste relevant sections from the manuscript (include quotes in quotation marks "like this" to indicate direct quotes from your manuscript), or elaborate on this item by providing additional information not in the ms, or briefly explain why the item is not applicable/relevant for your study

N/A

5-iv) Quality assurance methods

Provide information on quality assurance methods to ensure accuracy and quality of information provided [1], if applicable.

	1	2	3	4	5	
subitem not at all important	○	◉	○	○	○	essential

Clear selection

Does your paper address subitem 5-iv?

Copy and paste relevant sections from the manuscript (include quotes in quotation marks "like this" to indicate direct quotes from your manuscript), or elaborate on this item by providing additional information not in the ms, or briefly explain why the item is not applicable/relevant for your study

"we include feedback pages across all the interventions, at multiple points within modules, to elicit questions, concerns, and participant attitudes. Open-ended textboxes are available, along with clickable rating buttons. We also have an active process to catalog the feedback, change content when appropriate, and respond back to participants. Similarly, if a participant encounters a technical issue, the SMART toolbar has a dedicated button called, “Technical Help,” which allows participants within the intervention to send study staff reports of the issue. The SMART platform automatically codes the message with the participant’s browser, device, platform, and device operating system version."

5-v) Ensure replicability by publishing the source code, and/or providing screenshots/screen-capture video, and/or providing flowcharts of the algorithms used

Ensure replicability by publishing the source code, and/or providing screenshots/screen-capture video, and/or providing flowcharts of the algorithms used. Replicability (i.e., other researchers should in principle be able to replicate the study) is a hallmark of scientific reporting.

	1	2	3	4	5	
subitem not at all important	○	●	○	○	○	essential

Clear selection

Does your paper address subitem 5-v?

Copy and paste relevant sections from the manuscript (include quotes in quotation marks "like this" to indicate direct quotes from your manuscript), or elaborate on this item by providing additional information not in the ms, or briefly explain why the item is not applicable/relevant for your study

N/A

5-vi) Digital preservation

Digital preservation: Provide the URL of the application, but as the intervention is likely to change or disappear over the course of the years; also make sure the intervention is archived (Internet Archive, webcitation.org, and/or publishing the source code or screenshots/videos alongside the article). As pages behind login screens cannot be archived, consider creating demo pages which are accessible without login.

	1	2	3	4	5	
subitem not at all important	○	●	○	○	○	essential

Clear selection

Does your paper address subitem 5-vi?

Copy and paste relevant sections from the manuscript (include quotes in quotation marks "like this" to indicate direct quotes from your manuscript), or elaborate on this item by providing additional information not in the ms, or briefly explain why the item is not applicable/relevant for your study

N/A

5-vii) Access

Access: Describe how participants accessed the application, in what setting/context, if they had to pay (or were paid) or not, whether they had to be a member of specific group. If known, describe how participants obtained “access to the platform and Internet” [1]. To ensure access for editors/reviewers/readers, consider to provide a “backdoor” login account or demo mode for reviewers/readers to explore the application (also important for archiving purposes, see vi).

	1	2	3	4	5	
subitem not at all important	○	○	○	◉	○	essential

Clear selection

Does your paper address subitem 5-vii? *

Copy and paste relevant sections from the manuscript (include quotes in quotation marks "like this" to indicate direct quotes from your manuscript), or elaborate on this item by providing additional information not in the ms, or briefly explain why the item is not applicable/relevant for your study

"Completion of the baseline assessment triggers an automatic email inviting the participant to login to SMART by going to the website, resetting their password using their username, and then logging in to access the first tier of the intervention. All participants, regardless of demographic characteristics or responses to their baseline assessment, are given access to SSE."

5-viii) Mode of delivery, features/functionalities/components of the intervention and comparator, and the theoretical framework

Describe mode of delivery, features/functionalities/components of the intervention and comparator, and the theoretical framework [6] used to design them (instructional strategy [1], behaviour change techniques, persuasive features, etc., see e.g., [7, 8] for terminology). This includes an in-depth description of the content (including where it is coming from and who developed it) [1]," whether [and how] it is tailored to individual circumstances and allows users to track their progress and receive feedback" [6]. This also includes a description of communication delivery channels and – if computer-mediated communication is a component – whether communication was synchronous or asynchronous [6]. It also includes information on presentation strategies [1], including page design principles, average amount of text on pages, presence of hyperlinks to other resources, etc. [1].

	1	2	3	4	5	
subitem not at all important	○	○	○	●	○	essential

Clear selection

Does your paper address subitem 5-viii? *

Copy and paste relevant sections from the manuscript (include quotes in quotation marks "like this" to indicate direct quotes from your manuscript), or elaborate on this item by providing additional information not in the ms, or briefly explain why the item is not applicable/relevant for your study

SMART Sex Ed: "The first module covers sexual orientation and gender identity in detail. The differences between the two are identified, with both being further framed as continuous social constructs (e.g., what transgender means relative to non-binary; what distinguishes someone identifying as gay from pansexual; why people describe their sexual/gender identity on a continuum). “Coming out” is explained and participants are given tips for how to disclose sexual orientation or gender identity to family. Finally, community resources and LGBTQ-friendly organizations are suggested for participants who may want more specific help regarding understanding their sexual or gender identities.
The second module explores sexual behaviors (e.g., receptive anal sex), including how to minimize discomfort and maximize pleasure. Detailed discussions of sexual consent are provided, as well as an explanation of the sexual health rights of adolescents (e.g., a state-by-state map explaining laws about sexual health testing and access to services without parental consent).
The third and longest module introduces participants to biological and behavioral sexual health. While traditional topics like differences between bacterial and viral STIs are discussed in detail, this section elaborates on the sexual health needs of AMSM. For example, the role of lubrication during anal sex is explained as a protective factor when used with condoms; PrEP is described; relative differences in sexual risk behaviors are visually depicted using an HIV risk calculator; and how to find a friendly LGBTQ-oriented HIV/STI testing site is provided.
Finally, participants are introduced to the topic of healthy relationships in the fourth module. Different relationship configurations are described (e.g., being single, dating, being in multiple relationships) and the differences between monogamy and non-monogamy are explained. Suggestions for enacting direct communication about relationship expectations are given."

SMART Squad: "The educational modalities used are different in SS relative to SSE. This intervention relies on a scripted video soap opera delivered across the episodes. It features interactive activities that encourage participants to reflect on their motivations and help them build behavioral skills. SS also has a forum, where participants can post asynchronous messages to each other under topics like “breaking the mold and being yourself,” “best/worst dates you’ve had,” and “parents/guardians.” The forum has a topic called, “ask the Sexpert,” where participants can post questions, which are directly answered by study staff who [illegible]

provide health education but not medical advice. Finally, after episode 6, participants complete a goal setting activity where they select three prevention or risk-reduction goals to attempt to accomplish in the next month and the next three months. These goals include but are not limited to getting an HIV test, obtaining a condom, using condoms during every sexual encounter, and talking to a healthcare provider about PrEP. Once selected, the activity helps users think through how to overcome likely barriers by using suggested strategies to achieve the goals. In short, SS encourages participants to consider their own sexual identity, sexual health, and psychological challenges and then, identify the best ways to overcome them.

The video soap opera follows 4 main characters who are in geographically different high schools across the United States. These characters meet each other in an online space called “SMART Squad,” and become fast friends. They share with each other different sexual orientation, sexual behavior, and relationship problems they encounter in their daily lives and ask each other (and other characters) for advice. Participants follow their storylines as the characters make healthy and unhealthy decisions and learn from their successes and failures. In addition to the video soap opera, there are activities that conceptually and visually aligned with the videos. For example, one of the video characters is about to have sex for the first time. His older partner is pressuring him to have condomless sex, and the character does not know how to respond. At that moment in the video, an activity pops up for participants to help the character by rating potential “condom comebacks” as weak or strong. The strongest response from the activity is spoken by the character when the video restarts, enabling successful condom use.

These examples show how changes in motivations and behavioral skills are enacted throughout SS using interconnected video and activities. Peer norms and tension for change are instilled through the storylines and then skill-building exercises support self-reevaluation, stimulus control, and reinforcement management. In terms of specific content, episode 1 focuses on health, emotional, life, and physical needs as well as tips for dating. Episode 2 delves into the social and emotional consequences of HIV infection (e.g., stigma, disclosure) and shows how to have difficult conversations in a relationship (e.g., discussing infidelity with a main partner). Episode 3 is the longest episode and covers how to resist peer pressure to use drugs, how and when to choose to have anal sex with a partner, how to talk to health care providers about sexual health, HIV/STI testing, condom use norms, and behavioral and biomedical prevention strategies (e.g., PrEP). Episode 4 introduces participants to societal pressures around gender norms, features a condom demonstration, itemizes the steps to consider before meeting an unfamiliar/anonymous partner for sex, describes how to reinitiate condoms into a relationship, and shows how alcohol/drug use contributes to sexual risk behaviors. Episode 5 outlines the steps to take if condoms are not used or if the

condom breaks (i.e., post-exposure prophylaxis). It also shows how to negotiate condom use before sex with a partner. Finally, episode 6 concludes the main intervention by covering the intrinsic and extrinsic pressures to have sex, sexting, control/agency surrounding sex with partners, and overcoming barriers to obtaining condoms. This last episode also has the characters reiterate the overall importance of condoms, PrEP, and consistent HIV/STI testing.
The two boosters (i.e., episodes 7 and 8) do not introduce new concepts, but reinforce main themes from the first six episodes, continue the storyline of the characters several months later, and conclude the plotlines. The two boosters also serve as check-ins for participants regarding the goals they made after episode 6. Participants report back whether they have accomplished their goals. If they have, they are asked to select a new goal. If they have not, they are asked to provide reasons for not completing the goal and then SS provides them with additional strategies to help them."

SMART Sessions: "The four sessions focus on increasing motivation to engage in safer sex behaviors, including using condoms during sexual intercourse, receiving an HIV/STI test or creating a routine around testing, and PrEP use. The first session begins with introductions, an explanation of the overall timeline and content of Sessions, limits to confidentiality, and a “priorities” activity. This activity asks the participant to list the most important priorities in their lives and asks about the following five priorities and how they might fit in to the priorities that the participant has already listed: family, independence, sexuality, school, and health. The coach then asks the participant to select their top three priorities from the list and discuss how these priorities might be related to the decisions that they make around sexual health. This purpose of this activity is to consider how HIV prevention may fit in with the participant’s broader goals and values and to serve as a jumping off point for discussing the participant’s sexual health practices. At this point, the coach collaborates with the participant regarding which topic they would like to explore first – HIV prevention or HIV testing. Using Motivational Interviewing strategies, the coach works with the participant to identify changes that they might want to make to their sexual health plan and will encourage the participant to brainstorm ways in which they may begin to make those changes. Participants are asked to take into account past successes that they may have had in regard to sexual health. The first session ends with a summary of their discussions and by scheduling the second session. The second session mimics the first, but focuses on whichever topic was not previously addressed (HIV prevention or HIV testing). By the end of the first two sessions, the participant and coach have discussed both the topic areas, identified moments for potential behavior change regarding prevention and testing, and developed potential sexual health goals for consideration.
The third session takes a different direction by focusing on PrEP education and

PrEP navigation. The session begins with a review of sessions one and two and a recount of any successes or failures surrounding HIV prevention and/or testing. Following, the coach provides the participant with a brief educational overview of PrEP, including its usefulness and navigation options (i.e., who prescribes it; where to find providers). Together, the coach and participant explore ideas about whether PrEP might be a right fit, or identify future milestones for the participant that may signify it might be right to start PrEP (i.e., becoming sexually active; having multiple sex partners). If PrEP is a good choice for the participant, the coach and participant discuss strategies and goals to move the participant towards PrEP acquisition and use. The session ends with a review of PrEP and the coach answering any additional questions from the participant. If this is the final session, there is also a review of all the material covered in the previous sessions, a discussion regarding what sexual health resources are available to the participant, and the coach saying goodbye to the participant. The fourth session, for those designated in advance, (i.e., those actively engaging in condomless anal sex), begins with the participant describing progress made since initiating Sessions. The coach spends time highlighting the changes in the participant’s thinking and describes the progress that the coach perceives the participant has made. Together, they discuss obstacles to past-change and steps to take towards future-change regarding HIV prevention, testing, and if applicable, PrEP uptake. The coach works with the participant to identify commitment statements, which the participant should consider before enacting risk behaviors, if applicable. Goals are finalized and any concluding questions or concerns are answered before this last session is completed."

5-ix) Describe use parameters

Describe use parameters (e.g., intended “doses” and optimal timing for use). Clarify what instructions or recommendations were given to the user, e.g., regarding timing, frequency, heaviness of use, if any, or was the intervention used ad libitum.

	1	2	3	4	5	
subitem not at all important	○	○	◉	○	○	essential

Clear selection

Does your paper address subitem 5-ix?

Copy and paste relevant sections from the manuscript (include quotes in quotation marks "like this" to indicate direct quotes from your manuscript), or elaborate on this item by providing additional information not in the ms, or briefly explain why the item is not applicable/relevant for your study

Previously described by subitem 5-viii

5-x) Clarify the level of human involvement

Clarify the level of human involvement (care providers or health professionals, also technical assistance) in the e-intervention or as co-intervention (detail number and expertise of professionals involved, if any, as well as “type of assistance offered, the timing and frequency of the support, how it is initiated, and the medium by which the assistance is delivered”. It may be necessary to distinguish between the level of human involvement required for the trial, and the level of human involvement required for a routine application outside of a RCT setting (discuss under item 21 – generalizability).

	1	2	3	4	5	
subitem not at all important	○	○	◉	○	○	essential

Clear selection

Does your paper address subitem 5-x?

Copy and paste relevant sections from the manuscript (include quotes in quotation marks "like this" to indicate direct quotes from your manuscript), or elaborate on this item by providing additional information not in the ms, or briefly explain why the item is not applicable/relevant for your study

Previously described by subitem 5-viii

5-xi) Report any prompts/reminders used

Report any prompts/reminders used: Clarify if there were prompts (letters, emails, phone calls, SMS) to use the application, what triggered them, frequency etc. It may be necessary to distinguish between the level of prompts/reminders required for the trial, and the level of prompts/reminders for a routine application outside of a RCT setting (discuss under item 21 – generalizability).

	1	2	3	4	5	
subitem not at all important	○	○	◉	○	○	essential

Clear selection

Does your paper address subitem 5-xi? *

Copy and paste relevant sections from the manuscript (include quotes in quotation marks "like this" to indicate direct quotes from your manuscript), or elaborate on this item by providing additional information not in the ms, or briefly explain why the item is not applicable/relevant for your study

"....communications/reminders...." Given the length of the manuscript, it is impossible to get into automated and manual reminder system we have established with our staff.

5-xii) Describe any co-interventions (incl. training/support)

Describe any co-interventions (incl. training/support): Clearly state any interventions that are provided in addition to the targeted eHealth intervention, as ehealth intervention may not be designed as stand-alone intervention. This includes training sessions and support [1]. It may be necessary to distinguish between the level of training required for the trial, and the level of training for a routine application outside of a RCT setting (discuss under item 21 – generalizability.

	1	2	3	4	5	
subitem not at all important	(●)	( )	( )	( )	( )	essential

Clear selection

Does your paper address subitem 5-xii? *

Copy and paste relevant sections from the manuscript (include quotes in quotation marks "like this" to indicate direct quotes from your manuscript), or elaborate on this item by providing additional information not in the ms, or briefly explain why the item is not applicable/relevant for your study

N/A

6a) Completely defined pre-specified primary and secondary outcome measures, including how and when they were assessed

Does your paper address CONSORT subitem 6a? *

Copy and paste relevant sections from the manuscript (include quotes in quotation marks "like this" to indicate direct quotes from your manuscript), or elaborate on this item by providing additional information not in the ms, or briefly explain why the item is not applicable/relevant for your study

This is described in Table 1 in the manuscript.

6a-i) Online questionnaires: describe if they were validated for online use and apply CHERRIES items to describe how the questionnaires were designed/deployed

If outcomes were obtained through online questionnaires, describe if they were validated for online use and apply CHERRIES items to describe how the questionnaires were designed/deployed [9].

	1	2	3	4	5	
subitem not at all important	○	○	○	○	◉	essential

Clear selection

Does your paper address subitem 6a-i?

Copy and paste relevant sections from manuscript text

Yes, they were pilot tested rigorously but his was not discusses in the manuscript.

6a-ii) Describe whether and how “use” (including intensity of use/dosage) was defined/measured/monitored

Describe whether and how “use” (including intensity of use/dosage) was defined/measured/monitored (logins, logfile analysis, etc.). Use/adoption metrics are important process outcomes that should be reported in any ehealth trial.

	1	2	3	4	5	
subitem not at all important	◉	○	○	○	○	essential

Clear selection

Does your paper address subitem 6a-ii?

Copy and paste relevant sections from manuscript text

Previously described by subitem 5-viii

6a-iii) Describe whether, how, and when qualitative feedback from participants was obtained

Describe whether, how, and when qualitative feedback from participants was obtained (e.g., through emails, feedback forms, interviews, focus groups).

	1	2	3	4	5	
subitem not at all important	○	○	○	○	◉	essential

Clear selection

Does your paper address subitem 6a-iii?

Copy and paste relevant sections from manuscript text

"we include feedback pages across all the interventions, at multiple points within modules, to elicit questions, concerns, and participant attitudes. Open-ended textboxes are available, along with clickable rating buttons. We also have an active process to catalog the feedback, change content when appropriate, and respond back to participants. Similarly, if a participant encounters a technical issue, the SMART toolbar has a dedicated button called, "Technical Help," which allows participants within the intervention to send study staff reports of the issue. The SMART platform automatically codes the message with the participant's browser, device, platform, and device operating system version."

6b) Any changes to trial outcomes after the trial commenced, with reasons

Does your paper address CONSORT subitem 6b? *

Copy and paste relevant sections from the manuscript (include quotes in quotation marks "like this" to indicate direct quotes from your manuscript), or elaborate on this item by providing additional information not in the ms, or briefly explain why the item is not applicable/relevant for your study

N/A

7a) How sample size was determined

NPT: When applicable, details of whether and how the clustering by care provides or centers was addressed

7a-i) Describe whether and how expected attrition was taken into account when calculating the sample size

Describe whether and how expected attrition was taken into account when calculating the sample size.

	1	2	3	4	5	
subitem not at all important	○	◉	○	○	○	essential

Clear selection

Does your paper address subitem 7a-i?

Copy and paste relevant sections from manuscript title (include quotes in quotation marks "like this" to indicate direct quotes from your manuscript), or elaborate on this item by providing additional information not in the ms, or briefly explain why the item is not applicable/relevant for your study

This is described by Table 2 in the manuscript.

7b) When applicable, explanation of any interim analyses and stopping guidelines

Does your paper address CONSORT subitem 7b? *

Copy and paste relevant sections from the manuscript (include quotes in quotation marks "like this" to indicate direct quotes from your manuscript), or elaborate on this item by providing additional information not in the ms, or briefly explain why the item is not applicable/relevant for your study

N/A; we have not run interim analyses previously or at this time.

8a) Method used to generate the random allocation sequence

NPT: When applicable, how care providers were allocated to each trial group

Does your paper address CONSORT subitem 8a? *

Copy and paste relevant sections from the manuscript (include quotes in quotation marks "like this" to indicate direct quotes from your manuscript), or elaborate on this item by providing additional information not in the ms, or briefly explain why the item is not applicable/relevant for your study

Yes, see answer to 8b.

8b) Type of randomisation; details of any restriction (such as blocking and block size)

Does your paper address CONSORT subitem 8b? *

Copy and paste relevant sections from the manuscript (include quotes in quotation marks "like this" to indicate direct quotes from your manuscript), or elaborate on this item by providing additional information not in the ms, or briefly explain why the item is not applicable/relevant for your study

"Randomization to Treatment Arms
Non-responders to SSE are randomized to one of four ERs, which determines the interventions that a participant receives, and the order in which they occur. This assignment is done using stratified block randomization [73]. Through stratification, we avoid imbalance of pre-specified factors that may be related to the primary outcomes, and/or to the intervention delivery itself. We randomized within eight strata comprising all combinations of the following three binary factors: language preference (English or Spanish), rurality (living in an urban or rural zip code), and lifetime anal sex experience (any or none). Within each stratum, ERs were assigned using permuted block design with blocks of size four. This ensures that at any point during the study each ER assignment is protected against large imbalances in language preference, rurality, and sexual experience. The R package 'blockrand' [74] was used to create the randomization allocation table."

9) Mechanism used to implement the random allocation sequence (such as sequentially numbered containers), describing any steps taken to conceal the sequence until interventions were assigned

Does your paper address CONSORT subitem 9? *

Copy and paste relevant sections from the manuscript (include quotes in quotation marks "like this" to indicate direct quotes from your manuscript), or elaborate on this item by providing additional information not in the ms, or briefly explain why the item is not applicable/relevant for your study

See subitem 8b.

10) Who generated the random allocation sequence, who enrolled participants, and who assigned participants to interventions

Does your paper address CONSORT subitem 10? *

Copy and paste relevant sections from the manuscript (include quotes in quotation marks "like this" to indicate direct quotes from your manuscript), or elaborate on this item by providing additional information not in the ms, or briefly explain why the item is not applicable/relevant for your study

See subitem 8b

11a) If done, who was blinded after assignment to interventions (for example, participants, care providers, those assessing outcomes) and how

NPT: Whether or not administering co-interventions were blinded to group assignment

11a-i) Specify who was blinded, and who wasn’t

Specify who was blinded, and who wasn’t. Usually, in web-based trials it is not possible to blind the participants [1, 3] (this should be clearly acknowledged), but it may be possible to blind outcome assessors, those doing data analysis or those administering co-interventions (if any).

	1	2	3	4	5	
subitem not at all important	○	○	◉	○	○	essential

Clear selection

Does your paper address subitem 11a-i? *

Copy and paste relevant sections from the manuscript (include quotes in quotation marks "like this" to indicate direct quotes from your manuscript), or elaborate on this item by providing additional information not in the ms, or briefly explain why the item is not applicable/relevant for your study

Participants are not made aware of which arm they are a part of. They are also not aware of subsequent intervention tiers they may or may not be eligible for.

11a-ii) Discuss e.g., whether participants knew which intervention was the “intervention of interest” and which one was the “comparator”

Informed consent procedures (4a-ii) can create biases and certain expectations - discuss e.g., whether participants knew which intervention was the “intervention of interest” and which one was the “comparator”.

	1	2	3	4	5	
subitem not at all important	○	○	◉	○	○	essential

Clear selection

Does your paper address subitem 11a-ii?

Copy and paste relevant sections from the manuscript (include quotes in quotation marks "like this" to indicate direct quotes from your manuscript), or elaborate on this item by providing additional information not in the ms, or briefly explain why the item is not applicable/relevant for your study

Participants are not made aware of which arm they are a part of. They are also not aware of subsequent intervention tiers they may or may not be eligible for.

"To prevent participant anticipatory effects (i.e., misreporting with the intent to receive more or less treatment), they are not told the criteria for intervention response."

11b) If relevant, description of the similarity of interventions

(this item is usually not relevant for ehealth trials as it refers to similarity of a placebo or sham intervention to a active medication/intervention)

Does your paper address CONSORT subitem 11b? *

Copy and paste relevant sections from the manuscript (include quotes in quotation marks "like this" to indicate direct quotes from your manuscript), or elaborate on this item by providing additional information not in the ms, or briefly explain why the item is not applicable/relevant for your study

N/A - interventions are systematically different. See subitem 5-viii.

12a) Statistical methods used to compare groups for primary and secondary outcomes

NPT: When applicable, details of whether and how the clustering by care providers or centers was addressed

Does your paper address CONSORT subitem 12a? *

Copy and paste relevant sections from the manuscript (include quotes in quotation marks "like this" to indicate direct quotes from your manuscript), or elaborate on this item by providing additional information not in the ms, or briefly explain why the item is not applicable/relevant for your study

Yes - "Specifically, we will test the hypothesis (H1) of no difference in actual condom use or intentions/self-efficacy to use condoms in the SS group relative to the control group (SSE2.0) (see within Figure 1, letter A). To understand potential effects of the interventions on health disparities, we will test the hypothesis of no difference in actual condom use or intentions/self-efficacy to use condoms in the SS group relative to the control group separately within each of the six NIH-defined racial and ethnic categories (see H2–H7 in Table 2). Furthermore, we will test this hypothesis for the SS group relative to the control group among subjects: residing in non-rural areas (H9); residing in rural areas (H10); identified as low SES according to a family affluence scale (H11); identified as medium or high SES according to a family affluence scale (H12); and, with younger and older ages (H13). Because we offer all of SMART and its interventions in Spanish, we will also test the effectiveness of SS (in Spanish) specifically among Spanish speakers (H8) relative to the control group (SSE2.0 in Spanish).
We will test each of these hypotheses using a two-sided difference of proportions t-test. For age, we seek to enroll approximately equal numbers of each age, and we will test for a significant interaction between treatment (SS vs. SSE2.0) and age using a logistic regression model. We will use a Bonferroni multiplicity adjustment to ensure that the family-wise error rate of testing (H1)-(H13) is no greater than 0.05. Power calculations, displayed in Table 2, show that even after this multiplicity adjustment there is sufficient power to detect a moderate difference, (i.e., a Cohen's effect size of 0.52) [107], in the proportion of responders with 80% power at the proposed sample size within each subgroup considered. All power calculations were performed using the 'pwr' package in the R programming language. Table 2 shows that we have apportioned the Type I error inversely with the anticipated size of each subgroup thereby ensuring sufficient power in the smaller subgroups. Finally, to account for attrition, we inflate each group's sample size shown in Table 2 by 15% for a total proposed sample size of 1,878.
We will also conduct a series of exploratory (i.e., hypothesis-generating) comparisons between interventions applied to non-responders to SSE and SSE 2.0. First, we will compare the response rates at 9-months among those assigned to SS with those assigned to Sessions. This may provide evidence about whether the more intensive and costly Sessions is more effective than SS among those that did not respond to the control condition/SSE 2.0 (see letter B within Figure 1). Second, we will compare response rates at 9-months among non-responders to SS [illegible]

assigned to SS Booster 2-realtive to those assigned to Sessions. This will provide evidence about whether those who do not respond to SS will benefit from Sessions or if continued access to SS content would be sufficient (see letter C within Figure 1). Finally, among responders to SSE, we will compare response rates at 9-months among those assigned to SS relative to follow-up only. This will provide evidence about whether those that response to information-only, online HIV education intervention (e.g., SSE), will see additional benefits from SS (see letter D within Figure 1). Unlike the primary comparisons, secondary analyses will not involve statistical tests of significance but rather will consist of descriptive statistics, visualizations, and (un-adjusted for multiplicity) confidence intervals. These results will be reported as exploratory."

12a-i) Imputation techniques to deal with attrition / missing values

Imputation techniques to deal with attrition / missing values: Not all participants will use the intervention/comparator as intended and attrition is typically high in ehealth trials. Specify how participants who did not use the application or dropped out from the trial were treated in the statistical analysis (a complete case analysis is strongly discouraged, and simple imputation techniques such as LOCF may also be problematic [4]).

	1	2	3	4	5	
subitem not at all important	○	◉	○	○	○	essential

Clear selection

Does your paper address subitem 12a-i? *

Copy and paste relevant sections from the manuscript (include quotes in quotation marks "like this" to indicate direct quotes from your manuscript), or elaborate on this item by providing additional information not in the ms, or briefly explain why the item is not applicable/relevant for your study

We are not at this stage yet.

12b) Methods for additional analyses, such as subgroup analyses and adjusted analyses

Does your paper address CONSORT subitem 12b? *

Copy and paste relevant sections from the manuscript (include quotes in quotation marks "like this" to indicate direct quotes from your manuscript), or elaborate on this item by providing additional information not in the ms, or briefly explain why the item is not applicable/relevant for your study

Yes - "We will also conduct a series of exploratory (i.e., hypothesis-generating) comparisons between interventions applied to non-responders to SSE and SSE 2.0. First, we will compare the response rates at 9-months among those assigned to SS with those assigned to Sessions. This may provide evidence about whether the more intensive and costly Sessions is more effective than SS among those that did not respond to the control condition/SSE 2.0 (see letter B within Figure 1). Second, we will compare response rates at 9-months among non-responders to SS assigned to SS Booster 2-realtive to those assigned to Sessions. This will provide evidence about whether those who do not respond to SS will benefit from Sessions or if continued access to SS content would be sufficient (see letter C within Figure 1). Finally, among responders to SSE, we will compare response rates at 9-months among those assigned to SS relative to follow-up only. This will provide evidence about whether those that response to information-only, online HIV education intervention (e.g., SSE), will see additional benefits from SS (see letter D within Figure 1). Unlike the primary comparisons, secondary analyses will not involve statistical tests of significance but rather will consist of descriptive statistics, visualizations, and (un-adjusted for multiplicity) confidence intervals. These results will be reported as exploratory."

X26) REB/IRB Approval and Ethical Considerations [recommended as subheading under "Methods"] (not a CONSORT item)

X26-i) Comment on ethics committee approval

	1	2	3	4	5	
subitem not at all important	○	○	○	◉	○	essential

Clear selection

Does your paper address subitem X26-i?

Copy and paste relevant sections from the manuscript (include quotes in quotation marks "like this" to indicate direct quotes from your manuscript), or elaborate on this item by providing additional information not in the ms, or briefly explain why the item is not applicable/relevant for your study

"The Northwestern University IRB granted SMART a waiver of signed documentation of informed consent/assent, as well as a waiver of parental permission for participants under 18."

x26-ii) Outline informed consent procedures

Outline informed consent procedures e.g., if consent was obtained offline or online (how? Checkbox, etc.?), and what information was provided (see 4a-ii). See [6] for some items to be included in informed consent documents.

	1	2	3	4	5	
subitem not at all important	○	○	○	◉	○	essential

Clear selection

Does your paper address subitem X26-ii?

Copy and paste relevant sections from the manuscript (include quotes in quotation marks "like this" to indicate direct quotes from your manuscript), or elaborate on this item by providing additional information not in the ms, or briefly explain why the item is not applicable/relevant for your study

"Participants are routed to a consent page with four decisional capacity questions, which assess their comprehension of study tasks, risks, and benefits, as well as how to exit the study [72]. They also submit a username for study staff approval on the consent page. Usernames cannot have any personally identifying information (e.g., name, email). If they provide consent, study staff email and/or text prospective participants to set up a videochat to verify participant identity, review the study tasks, and answer any of their questions. During this 5-minute videochat, AMSM are also asked to explain back to study staff what they will be asked to do as a SMART participant. Finally, if a participant has submitted a username with personally identifying information, the study staff will work with the participant to revise the username while on the videochat."

X26-iii) Safety and security procedures

Safety and security procedures, incl. privacy considerations, and any steps taken to reduce the likelihood or detection of harm (e.g., education and training, availability of a hotline)

	1	2	3	4	5	
subitem not at all important	○	○	●	○	○	essential

Clear selection

Does your paper address subitem X26-iii?

Copy and paste relevant sections from the manuscript (include quotes in quotation marks "like this" to indicate direct quotes from your manuscript), or elaborate on this item by providing additional information not in the ms, or briefly explain why the item is not applicable/relevant for your study

This is done within the context on consent and the videochat/identification verification process.

RESULTS

13a) For each group, the numbers of participants who were randomly assigned, received intended treatment, and were analysed for the primary outcome

NPT: The number of care providers or centers performing the intervention in each group and the number of patients treated by each care provider in each center

Does your paper address CONSORT subitem 13a? *

Copy and paste relevant sections from the manuscript (include quotes in quotation marks "like this" to indicate direct quotes from your manuscript), or elaborate on this item by providing additional information not in the ms, or briefly explain why the item is not applicable/relevant for your study

N/A; these values are contingent on attrition and cannot be predicted at this time. No outcomes evaluations have been conducted at this time.

13b) For each group, losses and exclusions after randomisation, together with reasons

Does your paper address CONSORT subitem 13b? (NOTE: Preferably, this is shown in a CONSORT flow diagram) *

Copy and paste relevant sections from the manuscript (include quotes in quotation marks "like this" to indicate direct quotes from your manuscript), or elaborate on this item by providing additional information not in the ms, or briefly explain why the item is not applicable/relevant for your study

N/A; these values are contingent on attrition and cannot be predicted at this time. No outcomes evaluations have been conducted at this time.

13b-i) Attrition diagram

Strongly recommended: An attrition diagram (e.g., proportion of participants still logging in or using the intervention/comparator in each group plotted over time, similar to a survival curve) or other figures or tables demonstrating usage/dose/engagement.

	1	2	3	4	5	
subitem not at all important	○	◉	○	○	○	essential

Clear selection

Does your paper address subitem 13b-i?

Copy and paste relevant sections from the manuscript or cite the figure number if applicable (include quotes in quotation marks "like this" to indicate direct quotes from your manuscript), or elaborate on this item by providing additional information not in the ms, or briefly explain why the item is not applicable/relevant for your study

N/A; these values are contingent on attrition and cannot be predicted at this time. No outcomes evaluations have been conducted at this time.

14a) Dates defining the periods of recruitment and follow-up

Does your paper address CONSORT subitem 14a? *

Copy and paste relevant sections from the manuscript (include quotes in quotation marks "like this" to indicate direct quotes from your manuscript), or elaborate on this item by providing additional information not in the ms, or briefly explain why the item is not applicable/relevant for your study

As of June 30, 2020, 1,285 AMSM had completed all baseline assessment components and were considered enrolled in the study. Of those enrolled, 326 AMSM have completed their 12-month follow-up survey and have finished participating in SMART. We proposed enrollment of 1,878 AMSM, with recruitment concluding at the end of May 2020. The final sample will be diverse in terms of race/ethnicity, primary language spoken (i.e., English and Spanish), geographic region, socioeconomic status, and urban/rural location.

14a-i) Indicate if critical “secular events” fell into the study period

Indicate if critical “secular events” fell into the study period, e.g., significant changes in Internet resources available or “changes in computer hardware or Internet delivery resources”

	1	2	3	4	5	
subitem not at all important	○	◉	○	○	○	essential

Clear selection

Does your paper address subitem 14a-i?

Copy and paste relevant sections from the manuscript (include quotes in quotation marks "like this" to indicate direct quotes from your manuscript), or elaborate on this item by providing additional information not in the ms, or briefly explain why the item is not applicable/relevant for your study

While COVID19 had been occurring (since March 2020), we do not address it in the protocol manuscript because it did not at all change the implementation of the trial or the interventions. We will account for this in our findings when we conduct them.

14b) Why the trial ended or was stopped (early)

Does your paper address CONSORT subitem 14b? *

Copy and paste relevant sections from the manuscript (include quotes in quotation marks "like this" to indicate direct quotes from your manuscript), or elaborate on this item by providing additional information not in the ms, or briefly explain why the item is not applicable/relevant for your study

N/A

15) A table showing baseline demographic and clinical characteristics for each group

NPT: When applicable, a description of care providers (case volume, qualification, expertise, etc.) and centers (volume) in each group

Does your paper address CONSORT subitem 15? *

Copy and paste relevant sections from the manuscript (include quotes in quotation marks "like this" to indicate direct quotes from your manuscript), or elaborate on this item by providing additional information not in the ms, or briefly explain why the item is not applicable/relevant for your study

We cite the number recruited so far as a result but do not go into racial/ethnic differences at this time because those values will change as recruitment continues.

15-i) Report demographics associated with digital divide issues

In ehealth trials it is particularly important to report demographics associated with digital divide issues, such as age, education, gender, social-economic status, computer/Internet/ehealth literacy of the participants, if known.

	1	2	3	4	5	
subitem not at all important	○	◉	○	○	○	essential

Clear selection

Does your paper address subitem 15-i? *

Copy and paste relevant sections from the manuscript (include quotes in quotation marks "like this" to indicate direct quotes from your manuscript), or elaborate on this item by providing additional information not in the ms, or briefly explain why the item is not applicable/relevant for your study

N/A

16) For each group, number of participants (denominator) included in each analysis and whether the analysis was by original assigned groups

16-i) Report multiple “denominators” and provide definitions

Report multiple “denominators” and provide definitions: Report N’s (and effect sizes) “across a range of study participation [and use] thresholds” [1], e.g., N exposed, N consented, N used more than x times, N used more than y weeks, N participants “used” the intervention/comparator at specific pre-defined time points of interest (in absolute and relative numbers per group). Always clearly define “use” of the intervention.

	1	2	3	4	5	
subitem not at all important	○	○	◉	○	○	essential

Clear selection

Does your paper address subitem 16-i? *

Copy and paste relevant sections from the manuscript (include quotes in quotation marks "like this" to indicate direct quotes from your manuscript), or elaborate on this item by providing additional information not in the ms, or briefly explain why the item is not applicable/relevant for your study

This may be found in Table 2.

16-ii) Primary analysis should be intent-to-treat

Primary analysis should be intent-to-treat, secondary analyses could include comparing only “users”, with the appropriate caveats that this is no longer a randomized sample (see 18-i).

	1	2	3	4	5	
subitem not at all important	◉	○	○	○	○	essential

Clear selection

Does your paper address subitem 16-ii?

Copy and paste relevant sections from the manuscript (include quotes in quotation marks "like this" to indicate direct quotes from your manuscript), or elaborate on this item by providing additional information not in the ms, or briefly explain why the item is not applicable/relevant for your study

Your answer

17a) For each primary and secondary outcome, results for each group, and the estimated effect size and its precision (such as 95% confidence interval)

Does your paper address CONSORT subitem 17a? *

Copy and paste relevant sections from the manuscript (include quotes in quotation marks "like this" to indicate direct quotes from your manuscript), or elaborate on this item by providing additional information not in the ms, or briefly explain why the item is not applicable/relevant for your study

We do not present any outcomes yet due to the continued follow-up occurring within the trial. Not enough data has been collected yet.

17a-i) Presentation of process outcomes such as metrics of use and intensity of use

In addition to primary/secondary (clinical) outcomes, the presentation of process outcomes such as metrics of use and intensity of use (dose, exposure) and their operational definitions is critical. This does not only refer to metrics of attrition (13-b) (often a binary variable), but also to more continuous exposure metrics such as “average session length”. These must be accompanied by a technical description how a metric like a “session” is defined (e.g., timeout after idle time) [1] (report under item 6a).

	1	2	3	4	5	
subitem not at all important	○	◉	○	○	○	essential

Clear selection

Does your paper address subitem 17a-i?

Copy and paste relevant sections from the manuscript (include quotes in quotation marks "like this" to indicate direct quotes from your manuscript), or elaborate on this item by providing additional information not in the ms, or briefly explain why the item is not applicable/relevant for your study

We do not present any outcomes yet due to the continued follow-up occurring within the trial. Not enough data has been collected yet.

17b) For binary outcomes, presentation of both absolute and relative effect sizes is recommended

Does your paper address CONSORT subitem 17b? *

Copy and paste relevant sections from the manuscript (include quotes in quotation marks "like this" to indicate direct quotes from your manuscript), or elaborate on this item by providing additional information not in the ms, or briefly explain why the item is not applicable/relevant for your study

We do not present any outcomes yet due to the continued follow-up occurring within the trial. Not enough data has been collected yet.

18) Results of any other analyses performed, including subgroup analyses and adjusted analyses, distinguishing pre-specified from exploratory

Does your paper address CONSORT subitem 18? *

Copy and paste relevant sections from the manuscript (include quotes in quotation marks "like this" to indicate direct quotes from your manuscript), or elaborate on this item by providing additional information not in the ms, or briefly explain why the item is not applicable/relevant for your study

We do not present any outcomes yet due to the continued follow-up occurring within the trial. Not enough data has been collected yet.

18-i) Subgroup analysis of comparing only users

A subgroup analysis of comparing only users is not uncommon in ehealth trials, but if done, it must be stressed that this is a self-selected sample and no longer an unbiased sample from a randomized trial (see 16-iii).

	1	2	3	4	5	
subitem not at all important	○	●	○	○	○	essential

Clear selection

Does your paper address subitem 18-i?

Copy and paste relevant sections from the manuscript (include quotes in quotation marks "like this" to indicate direct quotes from your manuscript), or elaborate on this item by providing additional information not in the ms, or briefly explain why the item is not applicable/relevant for your study

We do not present any outcomes yet due to the continued follow-up occurring within the trial. Not enough data has been collected yet.

19) All important harms or unintended effects in each group

(for specific guidance see CONSORT for harms)

Does your paper address CONSORT subitem 19? *

Copy and paste relevant sections from the manuscript (include quotes in quotation marks "like this" to indicate direct quotes from your manuscript), or elaborate on this item by providing additional information not in the ms, or briefly explain why the item is not applicable/relevant for your study

No harm has been reported by our participants. We do not address within the manuscript.

19-i) Include privacy breaches, technical problems

Include privacy breaches, technical problems. This does not only include physical "harm" to participants, but also incidents such as perceived or real privacy breaches [1], technical problems, and other unexpected/unintended incidents. "Unintended effects" also includes unintended positive effects [2].

	1	2	3	4	5	
subitem not at all important	○	●	○	○	○	essential

Clear selection

Does your paper address subitem 19-i?

Copy and paste relevant sections from the manuscript (include quotes in quotation marks "like this" to indicate direct quotes from your manuscript), or elaborate on this item by providing additional information not in the ms, or briefly explain why the item is not applicable/relevant for your study

Your answer

19-ii) Include qualitative feedback from participants or observations from staff/researchers

Include qualitative feedback from participants or observations from staff/researchers, if available, on strengths and shortcomings of the application, especially if they point to unintended/unexpected effects or uses. This includes (if available) reasons for why people did or did not use the application as intended by the developers.

	1	2	3	4	5	
subitem not at all important	○	◉	○	○	○	essential

Clear selection

Does your paper address subitem 19-ii?

Copy and paste relevant sections from the manuscript (include quotes in quotation marks "like this" to indicate direct quotes from your manuscript), or elaborate on this item by providing additional information not in the ms, or briefly explain why the item is not applicable/relevant for your study

We do not present any feedback from our participants as that would be premature.

DISCUSSION

22) Interpretation consistent with results, balancing benefits and harms, and considering other relevant evidence

NPT: In addition, take into account the choice of the comparator, lack of or partial blinding, and unequal expertise of care providers or centers in each group

22-i) Restate study questions and summarize the answers suggested by the data, starting with primary outcomes and process outcomes (use)

Restate study questions and summarize the answers suggested by the data, starting with primary outcomes and process outcomes (use).

	1	2	3	4	5	
subitem not at all important	○	○	●	○	○	essential

Clear selection

Does your paper address subitem 22-i? *

Copy and paste relevant sections from the manuscript (include quotes in quotation marks "like this" to indicate direct quotes from your manuscript), or elaborate on this item by providing additional information not in the ms, or briefly explain why the item is not applicable/relevant for your study

No, because of the lack of data. Rather, we discuss the strengths and limitations of the study, its design, and its implementation.

22-ii) Highlight unanswered new questions, suggest future research

Highlight unanswered new questions, suggest future research.

	1	2	3	4	5	
subitem not at all important	○	●	○	○	○	essential

Clear selection

Does your paper address subitem 22-ii?

Copy and paste relevant sections from the manuscript (include quotes in quotation marks "like this" to indicate direct quotes from your manuscript), or elaborate on this item by providing additional information not in the ms, or briefly explain why the item is not applicable/relevant for your study

Due to size of the manuscript, we do not delve into specific avenues for future research. Rather, we discuss the potential of the current study.

20) Trial limitations, addressing sources of potential bias, imprecision, and, if relevant, multiplicity of analyses

20-i) Typical limitations in ehealth trials

Typical limitations in ehealth trials: Participants in ehealth trials are rarely blinded. Ehealth trials often look at a multiplicity of outcomes, increasing risk for a Type I error. Discuss biases due to non-use of the intervention/usability issues, biases through informed consent procedures, unexpected events.

	1	2	3	4	5	
subitem not at all important	○	○	○	●	○	essential

Clear selection

Does your paper address subitem 20-i? *

Copy and paste relevant sections from the manuscript (include quotes in quotation marks "like this" to indicate direct quotes from your manuscript), or elaborate on this item by providing additional information not in the ms, or briefly explain why the item is not applicable/relevant for your study

"Limitations

There are several limitations SMART faces in its current form and while we actively enroll AMSM. SMART is an eHealth intervention, which means that for SSE, SS, and SSE2.0, study staff are not present when participants access and move through intervention content. If participants have questions or concerns while viewing materials, there is no synchronously available moderator to help. Similarly, if participants encounter technical trouble while viewing any content, the onus is on the participant to contact study staff and report the issue. To counteract these potential issues, we include feedback pages across all the interventions, at multiple points within modules, to elicit questions, concerns, and participant attitudes. Open-ended textboxes are available, along with clickable rating buttons. We also have an active process to catalog the feedback, change content when appropriate, and respond back to participants. Similarly, if a participant encounters a technical issue, the SMART toolbar has a dedicated button called, "Technical Help," which allows participants within the intervention to send study staff reports of the issue. The SMART platform automatically codes the message with the participant's browser, device, platform, and device operating system version.

Participant attention during the intervention is another potential concern. During SSE, SS, and SSE2.0, how intently participants are focusing on the content cannot be precisely measured. Given other online (e.g., social media, television) and offline distractions (e.g., homework, chores, extracurricular activities), it may be possible that participants are focusing less on SMART content than if delivered in-person using a traditional modality like lecture or discussion. We do measure time-through-intervention; and while few participants appear to rush through the intervention (e.g., viewing for 10 minutes or less), overall focus may be inconsistent and an unmeasured individual participant difference. During Sessions, SMART coaches have anecdotally indicated several cases in which they suspected participants were multitasking using other apps while engaging in discussions. In these cases, coaches acknowledge that the participant may be distracted and attempt to refocus the individual or reschedule the session.

In addition to these operational limitations, there exists the larger issue of trying to test a sequential multiple assignment randomized trial intervention with such a young population. This sort of trial requires participants to engage with multiple interventions of varying intensities and lengths. More than 90% of participants will ultimately receive at least two interventions, if not three, over the [illegible]

course of 12 months. Considering many of these participants may not intrinsically be motivated towards, or interested in, sexual health education, this amount of content may exceed participant interest. Granted, months transpire between interventions; this remains a potential problem when working with adolescents who already are saturated with formal and informal education on a daily basis."

21) Generalisability (external validity, applicability) of the trial findings

NPT: External validity of the trial findings according to the intervention, comparators, patients, and care providers or centers involved in the trial

21-i) Generalizability to other populations

Generalizability to other populations: In particular, discuss generalizability to a general Internet population, outside of a RCT setting, and general patient population, including applicability of the study results for other organizations

	1	2	3	4	5	
subitem not at all important	○	◉	○	○	○	essential

Clear selection

Does your paper address subitem 21-i?

Copy and paste relevant sections from the manuscript (include quotes in quotation marks "like this" to indicate direct quotes from your manuscript), or elaborate on this item by providing additional information not in the ms, or briefly explain why the item is not applicable/relevant for your study

N/A; we are not prepared to discuss these aspects at this stage in the trial.

21-ii) Discuss if there were elements in the RCT that would be different in a routine application setting

Discuss if there were elements in the RCT that would be different in a routine application setting (e.g., prompts/reminders, more human involvement, training sessions or other co-interventions) and what impact the omission of these elements could have on use, adoption, or outcomes if the intervention is applied outside of a RCT setting.

	1	2	3	4	5	
subitem not at all important	○	◉	○	○	○	essential

Clear selection

Does your paper address subitem 21-ii?

Copy and paste relevant sections from the manuscript (include quotes in quotation marks "like this" to indicate direct quotes from your manuscript), or elaborate on this item by providing additional information not in the ms, or briefly explain why the item is not applicable/relevant for your study

N/A

OTHER INFORMATION

23) Registration number and name of trial registry

Does your paper address CONSORT subitem 23? *

Copy and paste relevant sections from the manuscript (include quotes in quotation marks "like this" to indicate direct quotes from your manuscript), or elaborate on this item by providing additional information not in the ms, or briefly explain why the item is not applicable/relevant for your study

Yes - Trial Registration: ClinicalTrials.gov Identifier NCT03511131; https://clinicaltrials.gov/ct2/show/NCT03511131

24) Where the full trial protocol can be accessed, if available

Does your paper address CONSORT subitem 24? *

Cite a Multimedia Appendix, other reference, or copy and paste relevant sections from the manuscript (include quotes in quotation marks "like this" to indicate direct quotes from your manuscript), or elaborate on this item by providing additional information not in the ms, or briefly explain why the item is not applicable/relevant for your study

The full trial protocol is not yet available; however, virtually all of the design and implementation may be viewed publicly at https://clinicaltrials.gov/ct2/show/NCT03511131

25) Sources of funding and other support (such as supply of drugs), role of funders

Does your paper address CONSORT subitem 25? *

Copy and paste relevant sections from the manuscript (include quotes in quotation marks "like this" to indicate direct quotes from your manuscript), or elaborate on this item by providing additional information not in the ms, or briefly explain why the item is not applicable/relevant for your study

Yes, in its unblinded form:
"Funding Source: All phases of this study were supported by an NIH grant, U01MD011281."

X27) Conflicts of Interest (not a CONSORT item)

X27-i) State the relation of the study team towards the system being evaluated

In addition to the usual declaration of interests (financial or otherwise), also state the relation of the study team towards the system being evaluated, i.e., state if the authors/evaluators are distinct from or identical with the developers/sponsors of the intervention.

	1	2	3	4	5	
subitem not at all important	○	○	◉	○	○	essential

Clear selection

Does your paper address subitem X27-i?

Copy and paste relevant sections from the manuscript (include quotes in quotation marks "like this" to indicate direct quotes from your manuscript), or elaborate on this item by providing additional information not in the ms, or briefly explain why the item is not applicable/relevant for your study

Yes.
"Conflict of Interest
No authors have conflicts of interest relevant to this article to disclose."

About the CONSORT EHEALTH checklist

As a result of using this checklist, did you make changes in your manuscript? *

- ○ yes, major changes
- ○ yes, minor changes
- ◉ no

What were the most important changes you made as a result of using this checklist?

N/A

How much time did you spend on going through the checklist INCLUDING making changes in your manuscript *

2 hours

As a result of using this checklist, do you think your manuscript has improved? *

- ( ) yes
- (x) no
- ( ) Other:

Would you like to become involved in the CONSORT EHEALTH group?

This would involve for example becoming involved in participating in a workshop and writing an "Explanation and Elaboration" document

- ( ) yes
- (x) no
- ( ) Other:

Clear selection

Any other comments or questions on CONSORT EHEALTH

The point of providing any of the above information, piece-meal, remains unclear to me. It seems a duplication of the time spent writing and working on the manuscript and its revisions.

STOP - Save this form as PDF before you click submit

To generate a record that you filled in this form, we recommend to generate a PDF of this page (on a Mac, simply select "print" and then select "print as PDF") before you submit it.

When you submit your (revised) paper to JMIR, please upload the PDF as supplementary file.

Don't worry if some text in the textboxes is cut off, as we still have the complete information in our database. Thank you!

Final step: Click submit !

Click submit so we have your answers in our database!

Submit

Never submit passwords through Google Forms.

This content is neither created nor endorsed by Google. Report Abuse - Terms of Service - Privacy Policy

Google Forms
